# Association of mineral and bone biomarkers with adverse cardiovascular outcomes and mortality in the German Chronic Kidney Disease (GCKD) cohort

**DOI:** 10.1038/s41413-023-00291-8

**Published:** 2023-10-20

**Authors:** Katharina Charlotte Reimer, Jennifer Nadal, Heike Meiselbach, Matthias Schmid, Ulla T. Schultheiss, Fruzsina Kotsis, Helena Stockmann, Nele Friedrich, Matthias Nauck, Vera Krane, Kai-Uwe Eckardt, Markus P. Schneider, Rafael Kramann, Jürgen Floege, Turgay Saritas, on behalf of the GCKD study investigators, Mario Schiffer, Hans-Ulrich Prokosch, Barbara Bärthlein, Andreas Beck, André Reis, Arif B. Ekici, Susanne Becker, Ulrike Alberth-Schmidt, Anke Weigel, Sabine Marschall, Eugenia Schefler, Gerd Walz, Anna Köttgen, Fruzsina Kotsis, Simone Meder, Erna Mitsch, Ursula Reinhard, Elke Schaeffner, Seema Baid-Agrawal, Kerstin Theisen, Kai Schmidt-Ott, Martin Zeier, Claudia Sommerer, Mehtap Aykac, Gunter Wolf, Rainer Paul, Antje Börner-Klein, Britta Bauer, Julia Raschenberger, Barbara Kollerits, Lukas Forer, Sebastian Schönherr, Hansi Weissensteiner, Peter Oefner, Wolfram Gronwald

**Affiliations:** 1 Department of Nephrology, Rheumatology, and Clinical Immunology, University Hospital RWTH Aachen, Aachen, Germany; 2 Institute of Experimental Medicine and Systems Biology, RWTH Aachen University, Aachen, Germany; 3 Institute for Cell and Tumor Biology, RWTH Aachen University, Aachen, Germany; 4 Institute of Medical Biometry, Informatics and Epidemiology, University Hospital of Bonn, Bonn, Germany; 5 Department of Nephrology and Hypertension, University of Erlangen-Nürnberg, Erlangen, Germany; 6 Institute of Genetic Epidemiology, Faculty of Medicine and Medical Center - University of Freiburg, Freiburg, Germany; 7 Department of Medicine IV – Nephrology and Primary Care, Faculty of Medicine and Medical Center - University of Freiburg, Freiburg, Germany; 8 Department of Nephrology and Medical Intensive Care, Charité-Universitätsmedizin Berlin, Berlin, Germany; 9 Department of Nephrology, University Medical Center Regensburg, Regensburg, Germany; 10 Institute of Clinical Chemistry and Laboratory Medicine, University Medicine Greifswald, Greifswald, Germany; 11 DZHK (German Centre for Cardiovascular Research), Partner Site Greifswald, University Medicine, Greifswald, Germany; 12 Department of Medicine I, Division of Nephrology, University Hospital Würzburg, Würzburg, Germany; 13 University of Erlangen-Nürnberg, Erlangen, Germany; 14 University of Freiburg, Freiburg, Germany; 15 Charité University Medicine Berlin, Berlin, Germany; 16 Hannover Medical School, Hannover, Germany; 17 University of Heidelberg, Heidelberg, Germany; 18 University of Jena, Jena, Germany; 19 University of Würzburg, Würzburg, Germany; 20 Medical University of Innsbruck, Institute of Genetic Epidemiology, Innsbruck, Austria; 21 University of Regensburg, Institute of Functional Genomics, Regensburg, Germany

**Keywords:** Calcium and phosphate metabolic disorders, Diseases

## Abstract

Mineral and bone disorder (MBD) in chronic kidney disease (CKD) is tightly linked to cardiovascular disease (CVD). In this study, we aimed to compare the prognostic value of nine MBD biomarkers to determine those associated best with adverse cardiovascular (CV) outcomes and mortality. In 5 217 participants of the German CKD (GCKD) study enrolled with an estimated glomerular filtration rate (eGFR) between 30–60 mL·min^−1^ per 1.73 m^2^ or overt proteinuria, serum osteoprotegerin (OPG), C-terminal fibroblast growth factor-23 (FGF23), intact parathyroid hormone (iPTH), bone alkaline phosphatase (BAP), cross-linked C-telopeptide of type 1 collagen (CTX1), procollagen 1 intact N-terminal propeptide (P1NP), phosphate, calcium, and 25-OH vitamin D were measured at baseline. Participants with missing values among these parameters (*n* = 971) were excluded, leaving a total of 4 246 participants for analysis. During a median follow-up of 6.5 years, 387 non-CV deaths, 173 CV deaths, 645 nonfatal major adverse CV events (MACEs) and 368 hospitalizations for congestive heart failure (CHF) were observed. OPG and FGF23 were associated with all outcomes, with the highest hazard ratios (HRs) for OPG. In the final Cox regression model, adjusted for CV risk factors, including kidney function and all other investigated biomarkers, each standard deviation increase in OPG was associated with non-CV death (HR 1.76, 95% CI: 1.35–2.30), CV death (HR 2.18, 95% CI: 1.50–3.16), MACE (HR 1.38, 95% CI: 1.12–1.71) and hospitalization for CHF (HR 2.05, 95% CI: 1.56–2.69). Out of the nine biomarkers examined, stratification based on serum OPG best identified the CKD patients who were at the highest risk for any adverse CV outcome and mortality.

## Introduction

Chronic kidney disease (CKD) is an increasing worldwide health burden associated with cardiovascular disease (CVD) and all-cause mortality.^1,2^ Traditional risk factors cannot fully explain the high incidence of fatal CVD events in CKD. Mineral and bone disorders (MBD) in CKD are common, and emerging evidence indicates a causal relationship between MBD and CVD through the progression of vascular calcification, fibrosis, and other mechanisms.^3^ Thus, the prognostic importance of bone and mineral parameters regarding cardiovascular (CV) outcomes and mortality is attracting increasing interest.^4,5^

Key markers of bone metabolism are osteoprotegerin (OPG), fibroblast growth factor-23 (FGF23), intact parathyroid hormone (iPTH), bone alkaline phosphatase (BAP), cross-linked C-telopeptide of type 1 collagen (CTX1), procollagen 1 intact N-terminal propeptide (P1NP), phosphate, calcium, and 25-OH vitamin D. OPG is a secreted member of the tumor necrosis factor receptor superfamily and protects against bone loss by inhibiting osteoclast activation.^6^ It has been associated with vascular calcification and mortality in the general population as well as in CKD.^7,8^ FGF23 is a phosphaturic hormone that is also capable of suppressing vitamin D metabolism. Elevated levels of FGF23 in CKD have been associated with CVD and mortality, although the mechanism of risk marked by FGF23 is not fully understood.^9^ BAP is essential for bone mineralization and has also been associated with vascular calcification and mortality in CKD.^10,11^ Collagen 1 is the most abundant component in human bone. P1NP is the amino terminal end of collagen 1 that is cleaved during collagen synthesis. As a result, it serves as a marker for collagen synthesis in bone turnover. CTX1, by contrast, is released upon the degradation of collagen 1 and consequently used as a bone resorption marker, for instance, in osteoporosis. CTX1 and P1NP are mainly studied in the field of osteoporosis, and it is not known whether these biomarkers can predict CVD and mortality in patients with CKD.^12^ Compared to those whose serum iPTH, calcium, and phosphate, 25-OH vitamin D levels fall outside of the recommended ranges in CKD, patients whose levels fall within the target ranges have a lower risk of mortality.^13,14^

Current limitations in determining the prognostic value of a biomarker to predict CVD and mortality are posed by study design, e.g., investigating only one or even a small number of biomarkers in a study weakens the comparability of different biomarkers for the same research question, as different studies vary methodologically. In addition, most studies are based on retrospective data and include challenges due to nonadjudicated endpoints and recall bias. In the present study, our aim was to generate better comparability between several bone metabolism biomarkers in CKD to identify those associated best with CVD events and mortality. Consequently, we measured the nine abovementioned biomarkers in baseline serum samples of CKD patients enrolled in the noninterventional multicenter German Chronic Kidney Disease (GCKD) study. Patients in this cohort were prospectively observed for a median time of 6.5 years, and all endpoints were adjudicated by an independent endpoint committee. Using this setting, we investigated the association of each biomarker with non-CV death, CV death, major adverse cardiac events (MACEs), and hospitalization due to congestive heart failure (CHF).

## Results

### Characteristics of the study cohort

The baseline characteristics of the cohort are shown in Table 1. The average BMI was in the overweight range, approximately 35% of the participants were diabetic, slightly more than a quarter reported the presence of CVD, and more than half of the study population were former or current smokers.

**Table 1:** Demographics and clinical parameters at baseline in the GCKD cohort (*n* = 4 246)

Age, years	60.3 ± 11.9
Male, *n*/%	2 609 (61.4)
Systolic BP/mmHg	139.4 ± 20.4
Diastolic BP/mmHg	79.1 ± 11.9
BMI/(kg·m^−2^)	29.9 ± 6.0
Diabetes, *n*/%	1 522 (35.8)
Previous CVD, *n*/%	1 123 (26.4)
Smoking	
Never, *n*/%	1 699 (40.1)
Former, *n*/%	1 835 (43.3)
Current, *n*/%	700 (16.5)
Unknown, *n*/%	12 (0.1)
eGFR/(mL·min^−1^ per 1.73 m^2^)	48.3 ± 17.5
UACR/(mg·g^−1^)	52.8 (9.9–385.0)
hsCRP/(mg·L^−1^)	2.3 (1.0–5.1)
Serum albumin/(mg·L^−1^)	38.7 (36.2–40.8)
LDL cholesterol/(mg·dL^−1^)	112.8 (88.1–141.9)
HDL cholesterol/(mg·dL^−1^)	47.9 (39.0–60.7)
BAP/(μg·L^−1^)	16.6 (13.1–21.3)
OPG/(pmol·L^−1^)	6.5 (5.0–8.3)
iPTH/(pg·mL^−1^)	38.7 (25.5–59.7)
FGF23/(pmol·L^−1^)	1.1 (0.6–2.2)
CTX1/(ng·mL^−1^)	0.3 (0.1–0.5)
Total calcium/(mmol·L^−1^)	2.27 (2.19–2.35)
Phosphate/(mmol·L^−1^)	1.11 (0.97–1.24)
25-OH vitamin D/(ng·mL^−1^)	23.0 (16.7–29.9)
P1NP/(ng·mL^−1^)	47.0 (34.9–65.5)
RASi, *n*/%	3 600 (84.8)
Statins, *n*/%	2 059 (48.5)
Beta blocker, *n*/%	2 360 (55.6)
Thrombocyte aggregation inhibitors, *n*/%	1 515 (35.7)
Aldosterone antagonists, *n*/%	343 (8.1)
Vitamin D therapy, *n*/%	1 320 (31.3)

Continuous variables are presented as the mean and standard deviation or median and interquartile range. Categorical variables are presented as absolute numbers and as percentages

*eGFR* estimated glomerular filtration rate, *UACR* urine albumin creatinine ratio, *hsCRP* high-sensitivity C-reactive protein, *LDL* low-density lipoprotein, *HDL* high-density lipoprotein, *BAP* bone alkaline phosphatase, *OPG* osteoprotegerin, *iPTH* intact parathyroid hormone, *FGF23* fibroblast growth factor-23, *CTX1* cross-linked C-telopeptide of type I collagen, *P1NP* procollagen I intact N-terminal, *CVD* cardiovascular disease, *BP* blood pressure, *BMI* body mass index, *RASi* renin-angiotensin system inhibitor

The demographic and clinical parameters stratified for the biomarker quintiles are shown in Tables S1–8. Given that serum OPG emerged as the most potent predictor of outcomes (see below), the distribution of demographic and clinical parameters stratified for OPG quintiles is shown separately in Table 2. Participants with higher OPG serum levels were older, had lower eGFR, more frequently had diabetes mellitus or CVD at baseline, had higher blood pressure, and used more medications (Table 2).

**Table 2:** Demographics and clinical parameters at baseline according to osteoprotegerin (OPG) quintiles in the GCKD cohort (*n* = 4 246)

	Osteoprotegerin/(pmol·L^−1^)				
Characteristics	Q1 (≤4.8)	Q2 (>4.8–≤5.9)	Q3 (>5.9–≤7.1)	Q4 (>7.1–≤8.7)	Q5 (>8.7)
*n*/%	850 (20.0)	849 (20.0)	849 (20.0)	850 (20.0)	848 (20.0)
Age/years	52.2 ± 13.4	56.6 ± 12.1	61.9 ± 9.9	64.5 ± 8.7	66.2 ± 8.4
Male, *n*/%	568 (13.4)	535 (12.6)	534 (12.6)	494 (11.6)	478 (11.3)
Systolic BP/mmHg	134.8 ± 17.6	137.6 ± 18.8	138.8 ± 19.8	143.2 ± 22.0	142.7 ± 22.1
Diastolic BP/mmHg	80.7 ± 11.4	80.4 ± 11.6	79.3 ± 11.9	78.7 ± 11.8	76.4 ± 12.3
BMI/(kg·m^–2^)	29.2 ± 5.4	29.7 ± 6.0	30.1 ± 6.2	30.7 ± 6.3	29.7 ± 5.9
Diabetes, *n*/%	183 (4.3)	223 (5.3)	289 (6.8)	389 (9.2)	438 (10.3)
Previous CVD, *n*/%	150 (3.5)	163 (3.8)	223 (5.3)	278 (6.5)	309 (7.3)
Smoking					
Never, *n*/%	340 (8.0)	334 (7.9)	335 (7.9)	341 (8.1)	349 (8.2)
Former, *n*/%	344 (8.1)	348 (8.2)	385 (9.1)	387 (9.1)	371 (8.8)
Current, *n*/%	164 (3.9)	165 (3.9)	127 (3.0)	120 (2.8)	124 (2.9)
Unknown, *n*/%	2 (0.0)	2 (0.0)	2 (0.0)	2 (0.0)	4 (0.1)
eGFR/(mL·min^–1^ per 1.73 m^2^)	53.8 ± 20.3	51.5 ± 17.8	47.4 ± 16.3	46.0 ± 15.7	42.6 ± 14.5
UACR/(mg·g^–1^)	72.4 (11.8–498.0)	54.3 (9.4–420.5)	31.3 (8.3–308.5)	46.8 (9.7–352.5)	62.6 (10.8–351.5)
hsCRP/(mg·L^−1^)	1.8 (0.8–4.0)	2.0 (0.9–4.7)	2.4 (1.1–5.4)	2.5 (1.2–5.2)	3.0 (1.4–6.7)
Serum albumin/(mg·L^−1^)	39.2 (36.7–41.3)	38.9 (36.6–41.0)	39.0 (36.5–41.0)	38.3 (36.1–40.6)	37.8 (35.3–40.2)
LDL cholesterol/(mg·dL^−1^)	115.1 (90.9–144.7)	115.8 (92.8–147.1)	114.3 (89.4–141.2)	112.0 (87.0–141.3)	106.6 (81.5–137.5)
HDL cholesterol/(mg·dL^−1^)	46.6 (37.9–59.4)	48.2 (39.1–59.7)	47.3 (39.2–59.8)	49.0 (39.7–60.8)	49.2 (39.3–63.4)
RASi, *n*/%	732 (17.2)	715 (16.8)	731 (17.2)	721 (17.0)	701 (16.5)
Statins, *n*/%	353 (8.4)	383 (9.1)	423 (10.0)	461 (10.9)	439 (10.4)
Beta blocker, *n*/%	391 (9.3)	415 (9.8)	506 (12.0)	532 (12.8)	516 (12.2)
Thrombocyte aggregation inhibitors, *n*/%	194 (4.6)	260 (6.2)	309 (7.3)	368 (8.7)	384 (9.1)
Aldosterone antagonists, *n*/%	58 (1.4)	54 (1.3)	67 (1.6)	80 (1.9)	84 (2.0)
Vitamin D therapy, *n*/%	260 (6.2)	239 (5.7)	255 (6.1)	272 (6.5)	294 (6.9)

Continuous variables are presented as the mean and standard deviation or median and interquartile range. Categorical variables are presented as absolute numbers and as percentages

*OPG* osteoprotegerin, *eGFR* estimated glomerular filtration rate, *UACR* urine albumin creatinine ratio, *hsCRP* high-sensitivity C-reactive protein, *LDL* low-density lipoprotein, *HDL* high-density lipoprotein, *BP* blood pressure, *CVD* cardiovascular disease, *BMI* body mass index, *RASi* renin-angiotensin system inhibitor

### Association between biomarker serum levels and non-CV death, CV death, MACE, and CHF events

**Fig. 1:**
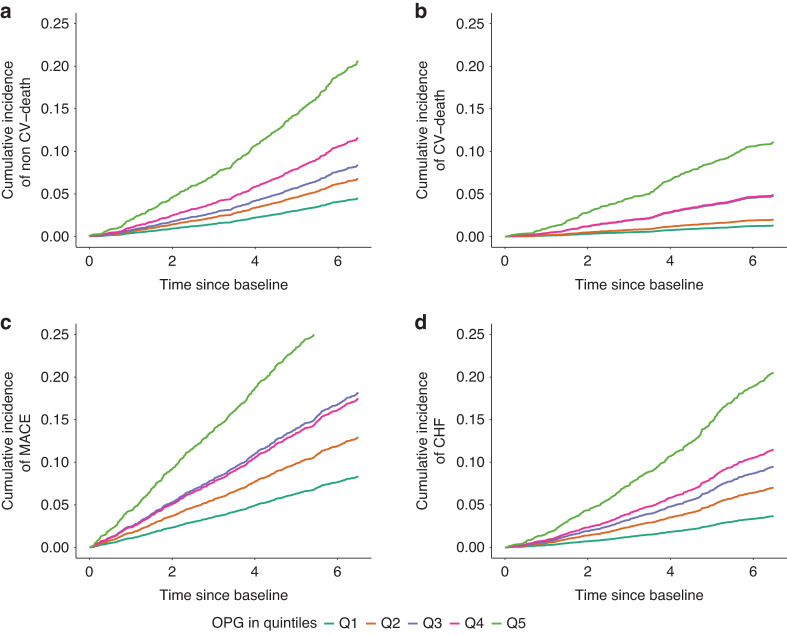
Cumulative incidence of (**a**) non-CV death, (**b**) CV death, (**c**) nonfatal MACE, and (**d**) hospitalization for congestive heart failure (CHF) according to OPG serum levels. Quintiles categorized as Q1, Q2, Q3, Q4, and Q5. Cumulative incidence derived from the unadjusted cause-specific hazard estimates (Model 1) by application of the Aalen-Johansen estimator. CV cardiovascular, OPG osteoprotegerin, MACE major adverse cardiovascular event

Increases in OPG, FGF23, and iPTH serum levels were associated with a higher risk for all outcomes in the univariate (Model 1) Cox regression analysis (Table 3, Tables S9–10). Figure 1 shows the unadjusted Aalen-Johansen cumulative incidence of non-CV death, CV death, MACE, and CHF hospitalizations for quintiles of OPG. In agreement with the results of the Cox regression analyses, the estimated incidence proportions became progressively greater with higher OPG levels for all outcomes studied. Higher serum levels of calcium and 25-OH vitamin D were associated with lower risk for all outcomes (Tables S11–S12). Higher BAP levels were associated with a higher risk for non-CV death, MACE, and CHF (Table S13), whereas higher CTX1 levels were associated with non-CV death, CV death, and MACE (Table S14). Furthermore, we found a weak association between higher serum phosphate levels and MACE (Table S15) and no association between P1NP levels and the outcomes (Supplementary Table S16).

**Table 3:** Association of osteoprotegerin (OPG) with different outcomes in three statistical models in the GCKD study

Outcomes		Univariate (Model 1)	Model 2	Model 3
		HR [95% CI limits]		
Non-CV death 387/4 245	HR per SD increase	3.73 [3.04; 4.57]	1.98 [1.53; 2.57]	1.76 [1.35; 2.30]
	Q1	1 (ref.)	1 (ref.)	1 (ref.)
	Q2	1.53 [1.00; 2.35]	1.18 [0.77; 1.83]	1.13 [0.73; 1.75]
	Q3	1.91 [1.26; 2.88]	1.10 [0.72; 1.69]	0.96 [0.63; 1.48]
	Q4	2.68 [1.81; 3.97]	1.27 [0.84; 1.91]	1.12 [0.74; 1.69]
	Q5	5.02 [3.47; 7.26]	1.86 [1.25; 2.76]	1.50 [1.00; 2.26]
CV death 173/4 245	HR per SD increase	4.44 [3.32; 5.95]	2.37 [1.65; 3.40]	2.18 [1.50; 3.16]
	Q1	1 (ref.)	1 (ref.)	1 (ref.)
	Q2	1.55 [0.70; 3.45]	1.23 [0.54; 2.76]	1.22 [0.54; 2.77]
	Q3	3.76 [1.87; 7.58]	2.16 [1.06; 4.40]	2.00 [0.97; 4.13]
	Q4	3.85 [1.91; 7.76]	1.67 [0.81; 3.43]	1.66 [0.80; 3.44]
	Q5	9.04 [4.67; 17.48]	3.26 [1.64; 6.49]	2.96 [1.47; 5.96]
MACE 645/4 244	HR per SD increase	2.71 [2.29; 3.21]	1.48 [1.20; 1.82]	1.38 [1.12; 1.71]]
	Q1	1 (ref.)	1 (ref.)	1 (ref.)
	Q2	1.59 [1.16; 2.17]	1.30 [0.94; 1.79]	1.26 [0.91; 1.75]
	Q3	2.30 [1.72; 3.09]	1.45 [1.07; 1.97]	1.42 [1.04; 1.94]
	Q4	2.20 [1.64; 2.97]	1.08 [0.79; 1.48]	1.04 [0.75; 1.43]
	Q5	4.09 [3.10; 5.40]	1.86 [1.37; 2.51]	1.70 [1.25; 2.32]
CHF 368/4 245	HR per SD increase	3.66 [2.96; 4.53]	2.19 [1.68; 2.85]	2.05 [1.56; 2.69]
	Q1	1 (ref.)	1 (ref.)	1 (ref.)
	Q2	1.94 [1.23; 3.08]	1.52 [0.95; 2.43]	1.48 [0.92; 2.37]
	Q3	2.66 [1.72; 4.12]	1.43 [0.91; 2.25]	1.35 [0.86; 2.13]
	Q4	3.25 [2.12; 4.99]	1.47 [0.94; 2.28]	1.38 [0.88; 2.16]
	Q5	6.14 [4.08; 9.22]	2.63 [1.72; 4.02]	2.43 [1.58; 3.76]

The results are presented as hazard ratios with 95% confidence intervals given in parentheses

Model 2: adjusted for age, sex, BMI, systolic blood pressure, LDL cholesterol, diabetes mellitus, eGFR, CVD, UACR, smoking, CRP, serum albumin, use of statins, use of RASis, use of thrombocyte aggregation inhibitors, use of beta blockers, use of aldosterone antagonists, and vitamin D therapy. Model 3: adjusted for parameters as in Model 2 plus BAP, calcium, phosphate, P1NP, CTX1, FGF23, 25-OH vitamin D, and iPTH

*OPG* osteoprotegerin, *ref.* reference, *HR* hazard ratio, *CI* confidence interval, *MACE* major adverse cardiac event, *CHF* hospitalization due to congestive heart failure, *BAP* bone alkaline phosphatase, *P1NP* procollagen I intact N-terminal propeptide, *CTX1* C-telopeptide of type 1 collagen, *FGF23* fibroblast growth factor-23, *iPTH* intact parathyroid hormone

In the second Cox regression model, we assessed the association between biomarker levels and outcomes after adjustment for known CV risk factors (see methods section). Higher OPG and FGF23 serum levels remained associated with a higher risk for all outcomes after the adjustment (Model 2 in Table 3 and Table S9). Higher iPTH levels remained associated with a higher risk for non-CV death and MACE but not with CV death and CHF (Table S10). No association was found between calcium levels and the outcomes, but higher 25-OH vitamin D levels were still associated with a lower risk for non-CV death, CV death, and MACE (Tables S11–12). After the adjustment, higher BAP and CTX1 serum levels remained associated with non-CV death (Tables S13–14). We did not find an association between higher serum phosphate levels and the outcomes (Table S15), but higher P1NP levels became significantly associated with CHF (Table S16).

**Fig. 2:**
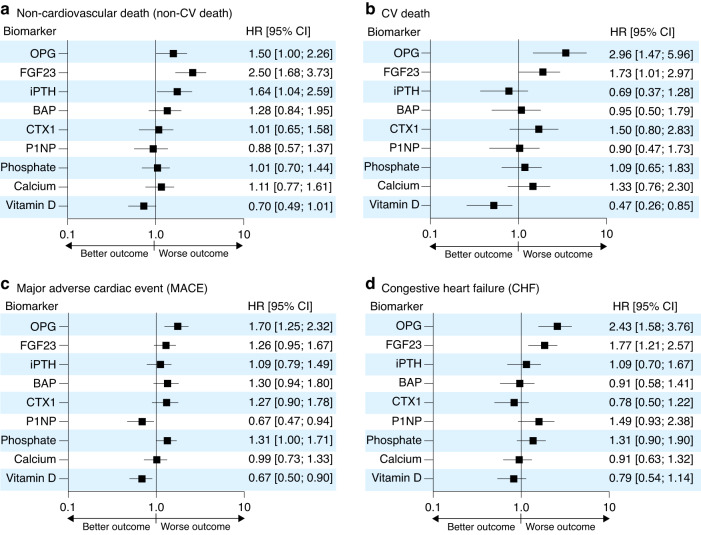
Risk of outcomes for Q5 vs. Q1 biomarker levels in a cohort of *n* = 4 246 patients enrolled in the German Chronic Kidney Disease Study (GCKD) in Model 3. The evaluated biomarkers OPG, FGF23, iPTH, BAP, CTX1, phosphate, calcium, and 25-OH Vitamin D were statistically associated with selected outcomes, including (**a**) non-CV death, (**b**) CV death, (**c**) MACE, and (**d**) CHF. Data of Model 3, which adjusted for demographic characteristics of the study population and for biomarker levels other than the analyzed one. CV cardiovascular, HR hazard ratio, CI confidence interval, MACE major adverse cardiac event, CHF hospitalization due to congestive heart failure, OPG osteoprotegerin, FGF23 fibroblast growth factor-23, iPTH intact parathyroid hormone, BAP bone alkaline phosphatase, CTX1 cross-linked C-telopeptide of type I collagen, P1NP procollagen I intact N-terminal

In the final model (Model 3), we additionally adjusted for all other measured bone biomarkers to further stress the prognostic power of the individual biomarkers for the outcomes (Fig. 2, Table 3 and Tables S9–16). In this model, OPG was the only biomarker consistently associated with all investigated outcomes. Per standard deviation increase in OPG, the risk for non-CV death, CV death, MACE and CHF hospitalizations increased by 76%, 118%, 38%, and 105%, respectively (Model 3 in Table 3). Similarly, Fig. 2 shows that compared to patients in the lowest OPG serum level quintile, those in OPG quintile 5 had a higher risk for non-CV death (+50%), CV death (+196%), MACE (+70%) and CHF (+143%). Higher FGF23 concentrations were associated with non-CV death (+150%), CV death (+73%), and CHF (+77%) but not MACE. Patients in iPTH quintile 5 had a +64% higher risk of suffering from non-CV death compared to those in iPTH quintile 1. In contrast, higher 25-OH vitamin D levels were still associated with a lower risk of CV death (HR 0.47, 95% CI: 0.26–0.85, Q5 vs. Q1) and MACE (HR 0.67, 95% CI: 0.50–0.90, Q5 vs. Q1). Higher phosphate levels were associated with MACEs (+31%), whereas patients in P1NP quintile 5 had a 33% lower risk for MACEs than those in quintile 1. No association was found between calcium, BAP, CTX1, and the outcomes. The full forest plots for each biomarker quintile in Model 3 are shown in Fig. S1.

## Discussion

We compared the association of the nine MBD parameters OPG, FGF23, iPTH, calcium, phosphate, BAP, CTX1, P1NP, and 25-OH vitamin D with adverse CV outcomes and death in patients with CKD. After extensive adjustment for several covariates and bone parameters, higher OPG levels demonstrated the strongest independent association with non-CV death, CV death, MACE, and hospitalization for CHF. Given that patients with CKD are at high risk for CVD, risk stratification based on biomarker levels seems of particular importance. This is especially important for patients with mild to moderate CKD (such as those represented in our cohort), in whom preventative measures are most effective. Although scholars have investigated the association of a single or a few biomarkers with adverse outcomes in previous studies, to our knowledge, none have compared the nine typical MBD biomarkers in a large prospective CKD cohort.

OPG serum levels increase with CKD stage,^15^ and OPG serum levels are associated with mortality in CKD patients.^16,17^ In the general population, higher OPG is associated with coronary events and heart failure hospitalizations.^18,19^ We provide the first evidence that this latter association is also valid in CKD patients. Despite the classical association of OPG with vascular calcification, the precise mechanisms through which OPG may contribute to increased CVD and mortality remain unclear. OPG knockout mice suffer from severe osteoporosis and increased calcification of the aortic wall.^20^ However, many clinical studies have shown a positive correlation between OPG levels and calcifications or future CV events.^21^

FGF23 regulates bone homeostasis and is overexpressed in inflammatory conditions. It activates profibrotic signaling and primes kidney fibroblasts via the transforming growth factor beta (TGFb) pathway.^22^ In several studies, FGF23 was linked to CKD progression, CV events, and all-cause mortality in CKD patients.^23,24^ We show that higher FGF23 was indeed associated with all outcomes after adjustment for demographic parameters, blood pressure, comorbidities, kidney function and medications. Nevertheless, once we made further adjustments for OPG and other bone parameters, the association between FGF23 and MACE disappeared. Overall, our data contribute to the growing body of evidence that identifies FGF23 as a marker for both CVD and mortality. However, compared to OPG, its association with these outcomes was weaker.

The progression of CKD is characterized by low serum vitamin D and calcium levels, whereas serum phosphate and iPTH levels increase, as confirmed in our study. We found higher 25-OH vitamin D levels to be associated with lower risk for the outcomes. This is in line with previous studies, showing inverse associations between circulating 25-OH vitamin D and CVD risk.^25^ It is worth noting that approximately 80% of our cohort exhibited either vitamin D insufficiency or deficiency. Therefore, our study suggests that adequate levels of vitamin D are linked to better outcomes, but excessive intake of vitamin D should be avoided, as the study design does not establish causality.

Previous studies showed U-shaped hazard ratio estimates for iPTH and mortality in dialysis patients.^13^ Similarly, high iPTH levels were previously associated with mortality in nondialysis-dependent CKD patients.^26^ Consistent with these reports, we found iPTH to be independently associated with non-CV death. Phosphate homeostasis is mainly regulated by FGF-23, its coreceptor klotho, vitamin D, and iPTH. Poor management of hyperphosphatemia in dialysis patients is associated with fatal CV events and mortality.^13,27^ As expected from patients with moderately impaired kidney function, only very few participants in our cohort had hyperphosphatemia (>1.45 mmol·L^–1^ or >4.5 mg·dL^–1^). Nevertheless, a few studies have shown that even in nondialysis-dependent CKD patients with serum phosphate levels in the normal ranges, higher phosphate is associated with CV events and mortality.^28,29^ However, other studies could not validate this association in individuals with nondialysis-dependent CKD.^30,31^ Similarly, we found no association between phosphate levels and mortality, but higher phosphate levels were associated with MACEs. Of note, patients from the earlier negative studies^30,31^ had fairly well-preserved kidney function; thus, differences in baseline characteristics may explain the divergent observations.

Serum BAP isoforms reflect bone turnover and comprise approximately 50% of total circulating alkaline phosphatase. One small study in 135 patients across all CKD stages found a correlation between BAP and CV events.^32^ The association of elevated serum BAP levels with mortality has been described in dialysis patients^11^ but not in patients with early CKD.^33^ Similarly, we found no association between BAP and the chosen outcomes.

CTX1 and P1NP are both markers of bone resorption and to date have mainly been studied in the context of osteoporosis. An association between CTX1 or P1NP and CV events or mortality has been described in the general population.^34–36^ In a recent study, CTX1 was reported to be associated with CV death and heart failure in patients with non–ST elevation-acute coronary syndrome.^36^ P1NP is considered a collagen accumulation and fibrosis marker,^37^ which accumulates with GFR decline.^38^ Higher urinary excretion of P1NP was associated with CV events and death in kidney transplant recipients.^39^ To the best of our knowledge, this is the first study to assess the association of these two biomarkers with CV outcomes in patients with CKD. We show that continuous increases in CTX1 levels are associated with non-CV death, MACE, and CHF after adjustment for traditional CV risk factors and despite adjustment for eGFR. P1NP was associated with hospitalization for CHF after adjustment for traditional risk factors, but this association disappeared after further adjustment for other bone biomarkers. Surprisingly, in the fully adjusted model, a categorical increase in P1NP became inversely associated with MACEs. More studies are needed to investigate the role of CTXI and P1NP as risk markers of CVD and mortality in CKD cohorts.

The limitations of our study include the circumstance that all analyzed biomarkers were measured only once at baseline. The participants were all enrolled in Germany with a predefined severity of CKD. Thus, it is uncertain whether the presented associations can be transferred to other populations with more advanced CKD or to countries with different ancestries. Another potential limitation of this study is that our C-terminal FGF23 ELISA recognized intact FGF23 as well as C-terminal FGF23 fragments, while an intact FGF23 ELISA may better represent the pure biological effect of FGF23.^40^ Nevertheless, given that kidney function or inflammation might have contributed to the heterogeneity in the strengths of associations of C-terminal vs. intact FGF23 with clinical outcomes in previous studies, we adjusted our models for kidney function and CRP to account for differences in C-terminal FGF23 clearance and cleavage.^41–43^ We have no measures of vascular calcification that are considered surrogate markers for hard outcomes. However, it is important to note that while vascular calcification may indicate the presence of CVD, it is not necessarily a direct cause of the outcomes. Therefore, our study focused on hard outcomes. Particular strengths are the large sample size, the prospective data acquisition, the ability to adjust for a large number of relevant risk factors and the central adjudication of outcomes, although we cannot exclude the possibility of residual confounding by unknown or unmeasured variables. One key aspect of this analysis is the direct comparability of the performance of nine biomarkers in the field of MBD within a large CKD cohort. Overall, studies of bone and mineral metabolism biomarkers investigating CV and non-CV outcomes in CKD patients are heterogeneous. This is mainly due to different study designs and varying characteristics of the study populations and outcomes.^44^ Studying multiple biomarkers for the same outcomes in a large CKD patient cohort can provide important insights into the performance and utility of different biomarkers and help to improve patient outcomes through more personalized care.

In conclusion, we analyzed nine CKD-MBD-associated biomarkers in a cohort of 4 246 participants enrolled in the GCKD study. Our approach, which involved analyzing multiple biomarkers in one study cohort, allowed for direct comparison of biomarker performance and overcame the limitations of comparing studies that only investigate one or a few biomarkers in each given patient cohort. We found that higher serum OPG levels consistently showed the strongest association with four major outcomes —CV death, non-CV death, MACE, and CHF—even after adjusting for other risk factors and bone biomarkers. The data—in aggregate—support a pathophysiological link between bone metabolism and CVD in CKD patients. In addition, it can help clinicians gain insights into a patient’s underlying health status and identify patients who are at high risk for CVD.

## Materials and methods

### Study design and participants

The GCKD study involved 5 217 participants of European ancestry aged 18 to 74 years with an eGFR of 30 to 60 mL·min^–1^ per 1.73 m^2^ (corresponding to CKD stage G3, A1-3) or an eGFR ≥60 mL·min^–1^ per 1.73 m^2^ in the presence of severely increased albuminuria (i.e., >300 mg·g^–1^ creatinine) (corresponding to CKD stages G1-2, A3).^45^ The GCKD study was approved by the ethics committees of all participating centers (Friedrich Alexander University of Erlangen-Nuremberg, University of Freiburg, Ludwig-Maximilians University of Munich, University of Hannover, Charité—Universitätsmedizin Berlin, University of Würzburg, RWTH Aachen, University of Jena, and Heidelberg University) and was registered in a national database for clinical studies (*Deutsches Register für Klinische Studien* (DRKS) 00003971). The main exclusion criteria included solid organ or stem cell transplantation, active malignancy within 24 months prior to screening, non-European ancestry, and severe heart failure (New York Heart Association (NYHA) Stage IV). Each study participant provided written informed consent.

### Biomarker serum level measurements

OPG, 25-OH vitamin D, P1NP, CTX1, iPTH, calcium, phosphate, FGF23, and BAP were measured in baseline serum samples from the GCKD study cohort that had been transported on dry ice and were stored at –80 °C. Measurements were performed at the Institute of Clinical Chemistry and Laboratory Medicine, Greifswald, Germany. OPG was measured by sandwich enzyme-linked immunosorbent assay (ELISA, Biomedica Immunoassays, Vienna, Austria, Cat# BI-20403) with a limit of detection (LOD) of 5.4 pg·mL^–1^ and an intra-assay variation ≤3%. C-terminal FGF23 was measured by ELISA (Biomedica Immunoassays; Cat# BI-20702) with an LOD of 0 pmol·L^–1^ + 3 standard deviations (SD) of 0.07 pmol·L^–1^ and an intra-assay variation ≤8%. 25-OH Vitamin D (LOD of 2.4 ng·mL^–1^ and an intra-assay variation of 4.6%–6.2%), P1NP (LOD < 1.0 ng·mL^–1^, intra-assay variation 2.6%–3.0%), iPTH (LOD 2.5 pg·mL^–1^, intra-assay variation 1.1%–6.3%), CTX1 (LOD 0.023 ng·mL^–1^, intra-assay variation 2.1%–4.9%), and BAP (LOD 0.4 μg·L^–1^, intra-assay variation 1.4%–2.0%) were measured by chemiluminescence assay on an IDS-iSYS platform (Immunodiagnostic Systems, Frankfurt, Germany). Reagents and standards were used as recommended by the manufacturers.

In addition, a standardized set of biomarkers was measured in a central certified laboratory using standardized protocols (Synlab, Germany).^46^ Calcium and phosphate were measured on a clinical chemistry platform (Cobas, Roche Diagnostics, Rotkreuz, Switzerland). Baseline serum and urinary creatinine were quantified using an IDMS traceable methodology (Creatinine plus, Roche Diagnostics, Rotkreuz, Switzerland). GFR was then estimated using the 2009 creatinine-based CKD Epidemiology Collaboration formula. Baseline urinary and serum albumin as well as low-density lipoprotein (LDL) cholesterol, high-density lipoprotein (HDL) cholesterol, and high-sensitive C-reactive protein (hsCRP) were quantified using a turbidimetric method (Tina-quant, on Roche/Hitachi MODULAR P platform (Roche Diagnostics, Rotkreuz, Switzerland). The urine albumin creatinine ratio (UACR) was calculated from the measured urinary albumin and urinary creatinine (mg·g^–1^).

### Outcome assessment

During follow-up, patients were subjected to yearly interviews by trained personnel in alternating phone visits and face-to-face interactions. In this structured interview setting, data on any hospitalizations or clinical events were recorded. The collected data were verified by discharge and outpatient letters authored by the respective physician(s) responsible for the patients’ treatments. These reports were further subjected to extraction of outcomes according to a prespecified endpoint catalog by independent physicians (endpoint adjudication committee). Eventual deaths of study participants were confirmed by obtaining death certificates from civil registry offices whenever possible. The outcomes analyzed in this report include non-CV death, CV death (including death due to myocardial infarction, coronary artery disease, death due to a cerebrovascular event, sudden cardiac death, and death due to other cardiac causes, including death due to pulmonary embolism, decompensated heart failure, cardiac valve disease, or pulmonary embolism), nonfatal major adverse cardiac events (MACE, i.e., nonfatal myocardial infarction and nonfatal ischemic or hemorrhagic stroke), and hospitalization for CHF.

### Statistical analysis of the clinical data

Participants who had missing values for bone and mineral biomarkers were excluded from the analysis (*n* = 917). Thus, the analysis data included 4 246 participants. The participants included in our analysis had characteristics comparable to those of the total GCKD study population (Fig. S2). We used mean values and standard deviations for normally distributed variables and median values and interquartile ranges for nonnormally distributed variables to describe the overall population and across the calcium, phosphate, P1NP, CTX1, OPG, FGF23, BAP, 25-OH vitamin D, and iPTH quintiles. Values for categorical variables are presented as frequency distributions with percentages. If patients failed to complete the 6.5-year follow-up period, censoring was performed at the time of the last follow-up, e.g., when participants left the study due to loss to follow-up or refused to further participate in the study. Patients were censored at death if it was not part of the outcome of interest; thus, all hazard estimates obtained from our models were cause specific. Data extraction from the main GCKD study database was performed in February 2021. At this time, 188 (2.3%) participants had been lost to follow-up, and 311 (5.9%) participants had prematurely left the study. All data collected during their active participation were used for this analysis.

We used Cox proportional hazard models to analyze the associations of OPG, FGF23, BAP, calcium, phosphate, P1NP, CTX1, iPTH, and 25-OH vitamin D with non-CV death, CV death, MACE, and hospitalization due to CHF during follow-up. We assessed each outcome with three statistical models. First, we applied a univariate model to OPG, FGF23, BAP, calcium, phosphate, P1NP, CTX1, iPTH and 25-OH vitamin D alone (Model 1). The subsequent multivariate model (Model 2) was additionally adjusted for traditional risk factors for CVD, including age, gender, body mass index (BMI), systolic blood pressure, presence of diabetes mellitus, history of smoking, preexisting CVD, laboratory parameters including LDL cholesterol, eGFR, UACR, hsCRP, serum albumin, and relevant medication, including use of statins, renin-angiotensin system inhibitors (RASi), anti-platelet aggregation agents, beta blockers, vitamin D and analogs supplementation, and mineralocorticoid receptor antagonists. The selection of variables adjusted for in Model 2 was also based on the differences in the clinical characteristics between the quintiles of the different biomarkers provided below. In multivariate Model 3, we additionally corrected for all other bone and mineral biomarkers included in this study. The resulting hazard ratios (HRs) are presented with 95% confidence intervals (CIs). Statistical analysis was performed with SAS version 9.4 (SAS Institute Inc., Cay, NC, USA). Data were visualized with SAS, R version 4.2.2, and GraphPad Prism version 9 (GraphPad Software Inc., San Diego, CA, USA).

### Supplementary information

Supplemental Material
