# Supplementary material for: Association of mineral and bone biomarkers with adverse cardiovascular outcomes and mortality in the German Chronic Kidney Disease (GCKD) cohort
Source: Bone Res. 2023 Oct 20;11:52. doi: 10.1038/s41413-023-00291-8 (PMC10587182; doi:10.1038/s41413-023-00291-8)
Supplement: Supplementary file 1 — Supplemental Material [file 41413_2023_291_MOESM1_ESM.pdf]

Association of mineral and bone biomarkers with adverse cardiovascular outcomes and mortality in the German Chronic Kidney Disease (GCKD) cohort

Supplementary Data

Table S1. Demographics and clinical parameters at baseline according to FGF23 quintiles (n=4246).

Table S2. Demographics and clinical parameters at baseline according to intact parathyroid hormone (iPTH) quintiles (n=4246).

Table S3. Demographics and clinical parameters at baseline according to bone alkaline phosphatase (BAP) quintiles (n=4246).

Table S4. Demographics and clinical parameters at baseline according to C-Telopeptide of Type I Collagen (CTX1) quintiles (n=4246).

Table S5. Demographics and clinical parameters at baseline according to Procollagen I Intact N-terminal (P1NP) quintiles (n=4246).

Table S6. Demographics and clinical parameters at baseline according to phosphate quintiles (n=4246).

Table S7. Demographics and clinical parameters at baseline according to calcium quintiles (n=4246).

Table S8. Demographics and clinical parameters at baseline according to 25-OH Vitamin D quintiles (n=4246).

Table S9. Association of fibroblast growth factor 23 (FGF23) with different outcomes.

Table S10. Association of intact parathormone (iPTH) with different outcomes.

Table S11. Association of serum calcium levels with different outcomes.

Table S12. Association of 25-OH Vitamin D with different outcomes.

Table S13. Association of bone alkaline phosphatase (BAP) with different outcomes.

Table S14. Association of C-telopeptide of type I collagen (CTX1) with different outcomes.

Table S15. Association of serum phosphate levels with different outcomes.

Table S16. Association of procollagen I intact N-terminal (P1NP) with different outcomes.

Figure S1. Risk of outcomes for each biomarker quintiles in model 3.

Figure S2. Radar plot for GCKD data.

Table S1. Demographics and clinical parameters at baseline according to iFGF23 quintiles (n=4246).

FGF23 (ng/ml)	Q1 (≤0.5 pmol/l)	Q2 (>0.5 - ≤0.9 pmol/l)	Q3 (>0.9 - ≤01.44 pmol/l)	Q4 (>1.44 - ≤2.58 pmol/l)	Q5 (>2.58 pmol/l)
N (%)	853 (20.1)	854 (20.1)	848 (20.0)	842 (19.8)	849 (20.0)
Demographics					
Age, years	59.5 ± 11.7	59.1 ± 12.0	60.0 ± 12.1	61.5 ± 11.2	61.4 ± 12.1
Male, N (%)	542 (12.8)	534 (12.6)	512 (12.1)	504 (11.9)	517 (12.2)
Laboratory Measures					
eGFR (ml/min per 1.73 m^2)	52.6 ± 17.6	52.5 ± 18.0	50.2 ± 18.7	44.8 ± 14.8	41.1 ± 15.1
UACR (mg/g)	42.6 (8.4-295.9)	41.1 (8.7-307.3)	58.2 (9.7-450.6)	52.3 (10.0-468.4)	76.3 (12.9-490.9)
hsCRP (mg/l)	1.8 (0.9-3.8)	1.9 (0.9-4.2)	2.3 (1.0-5.1)	2.6 (1.1-5.4)	3.4 (1.5-7.6)
Serum albumin (mg/l)	39.1 (36.7-41.2)	39.1 (37.0-41.2)	38.7 (36.6-40.8)	38.3 (35.8-40.5)	37.6 (35.1-40.2)
LDL cholesterol (mg/dl)	119.5 (94.1-146.8)	121.5 (93.5-147.1)	110.9 (88.6-141.1)	110.6 (85.7-139.8)	103.4 (81.0-130.1)
HDL cholesterol (mg/dl)	51.1 (42.2-63.6)	49.3 (40.1-62.0)	48.7 (39.6-61.4)	46.9 (37.9-59.2)	44.0 (36.1-55.1)
CVD risk factors					
Systolic BP (mmHg)	138.9 ± 19.3	139.8 ± 19.6	140.4 ± 20.5	140.8 ± 21.1	137.3 ± 21.3
Diastolic BP (mmHg)	80.3 ± 11.6	80.1 ± 11.5	80.4 ± 11.7	78.5 ± 12.0	76.2 ± 12.5
BMI (kg/m^2)	28.9 ± 5.2	29.2 ± 5.3	30.0 ± 5.9	30.4 ± 6.2	30.9 ± 7.0
Diabetes, N (%)	239 (5.6)	247 (5.8)	277 (6.5)	353 (8.3)	406 (9.6)
Previous CVD, N (%)	164 (3.9)	175 (4.1)	212 (5)	240 (5.7)	332 (7.8)
Smoking history					
never, N (%)	384 (9.1)	372 (8.8)	348 (8.2)	333 (7.9)	262 (6.2)
former, N (%)	374 (8.8)	363 (8.6)	357 (8.4)	359 (8.5)	382 (9.0)
current, N (%)	94 (2.2)	118 (2.8)	137 (3.2)	149 (3.5)	202 (4.8)
unknown, N	1 (0.0)	1 (0.0)	6 (0.1)	1 (0.0)	3 (0.0)
Relevant Medication					
RASi, N (%)	706 (16.6)	726 (17.1)	733 (17.3)	727 (17.1)	708 (16.7)
Statins, N (%)	353 (8.4)	379 (9.0)	423 (10)	454 (10.8)	450 (10.7)
Betablocker, N (%)	410 (9.7)	438 (10.4)	438 (10.4)	507 (12.0)	567 (13.4)
Aldosterone antagonists, N (%)	42 (1.0)	49 (1.2)	74 (1.8)	77 (1.8)	101 (2.4)
Thrombocyte aggregation inhibitors, N (%)	251 (6.0)	309 (7.3)	301 (7.1)	340 (8.1)	314 (7.4)
Vitamin D therapy, N (%)	219 (5.2)	246 (5.8)	250 (5.9)	262 (6.2)	343 (8.1)

Continuous variables are presented as mean and standard deviation or median and interquartile range. Categorical variables are presented as absolute numbers and as percentage of overall study population. iFGF23, intact fibroblast growth factor 23; eGFR, estimated glomerular filtration rate; UACR, urine albumin creatinine ratio; hsCRP, high-sensitivity C-reactive protein; LDL, low density lipoprotein; HDL, high density lipoprotein; BP, blood pressure; CVD, cardiovascular disease; BMI; body mass index, RASi; renin angiotensin system inhibitor.

Table S2. Demographics and clinical parameters at baseline according to intact parathyroid hormone (iPTH) quintiles (n=4246).

iPTH (pg/ml)	Q1 (≤22.8 pg/ml)	Q2 (>22.8 – 33.2≤ pg/ml)	Q3 (>33.2 – 45.9≤ pg/ml)	Q4 (>45.9 – 66.1≤ pg/ml)	Q5 (>66.1 pg/ml)
N (%)	852 (20.1)	856 (20.2)	844 (19.9)	846 (19.9)	848 (20.0)
Demographics					
Age, years	55.0 ± 13.9	60.1 ± 11.5	61.5 ± 10.9	62.5 ± 10.4	62.5 ± 10.6
Male, N (%)	488 (11.5)	516 (12.2)	525 (12.4)	525 (12.4)	555 (13.1)
Laboratory Measures					
eGFR (ml/min per 1.73 m^2)	60.6 ± 21.7	51.9 ± 16.6	47.2 ± 13.6	44.4 ± 13.6	37.3 ± 10.9
UACR (mg/g)	65.8 (9.7-559.4)	31.7 (7.2-266.5)	41.2 (8.5-303.7)	52.3 (10.7-353.7)	81.8 (15.3-551.8)
hsCRP (mg/l)	1.8 (0.9-4.2)	2.0 (0.9-4.6)	2.4 (1.0-4.9)	2.5 (1.2-5.5)	2.9 (1.3-6.6)
Serum albumin (mg/l)	38.9 (36.0-41.1)	38.9 (35.5-40.9)	38.9 (36.7-40.9)	38.4 (36.1-40.6)	38.1 (35.6-40.6)
LDL cholesterol (mg/dl)	121.9 (96.4-149.4)	115.8 (90.1-143.4)	113.2 (88.7-141.9)	108.5 (85.2-137.7)	103.6 (81.8-136.4)
HDL cholesterol (mg/dl)	51.4 (41.2-65.9)	50.8 (40.9-63.8)	47.2 (38.8-59.1)	46.9 (37.5-59.1)	45.1 (37.8-54.8)
CVD risk factors					
Systolic BP (mmHg)	136.3 ± 19.5	138.5 ± 19.2	139.7 ± 20.3	140.8 ± 20.7	141.9 ± 21.7
Diastolic BP (mmHg)	79.3 ± 11.2	79.5 ± 11.5	79.0 ± 11.7	78.7 ± 12.2	79.0 ± 12.9
BMI (kg/m^2)	28.6 ± 5.8	29.3 ± 5.3	29.8 ± 5.9	30.8 ± 6.2	31.0 ± 6.3
Diabetes, N (%)	239 (5.6)	281 (6.6)	303 (7.1)	337 (7.9)	362 (8.5)
Previous CVD, N (%)	145 (3.4)	184 (4.3)	225 (5.3)	256 (6.0)	313 (7.4)
Smoking history					
never, N (%)	336 (7.9)	338 (8.0)	372 (8.8)	327 (7.7)	326 (7.7)
former, N (%)	326 (7.7)	373 (8.8)	350 (8.3)	402 (9.5)	384 (9.1)
current, N (%)	187 (4.4)	143 (3.4)	120 (2.8)	116 (2.7)	134 (3.2)
unknown, N	3 (0.0)	2 (0.0)	2 (0.0)	1 (0.0)	4 (0.1)
Relevant Medication					
RASi, N (%)	694 (16.3)	729 (17.2)	717 (16.9)	721 (17.0)	739 (17.4)
Statins, N (%)	346 (8.2)	375 (8.9)	391 (9.3)	453 (10.7)	494 (11.7)
Betablocker, N (%)	340 (8.1)	446 (10.6)	479 (11.4)	525 (12.4)	570 (13.5)
Aldosterone antagonists, N (%)	59 (1.4)	63 (1.5)	66 (1.6)	72 (1.7)	83 (2.0)
Thrombocyte aggregation inhibitors, N (%)	236 (5.6)	279 (6.6)	309 (7.3)	345 (8.2)	346 (8.2)
Vitamin D therapy, N (%)	272 (6.5)	253 (6)	250 (5.9)	249 (5.9)	295 (7)

Continuous variables are presented as mean and standard deviation or median and interquartile range. Categorical variables are presented as absolute numbers and as percentage of overall study population. iPTH, intact parathormone, eGFR, estimated glomerular filtration rate; UACR, urine albumin creatinine ratio; hsCRP, high-sensitivity C-reactive protein; LDL, low density lipoprotein; HDL, high density lipoprotein; BP, blood pressure; CVD, cardiovascular disease; BMI; body mass index, RASi; renin angiotensin system inhibitor.

Table S3. Demographics and clinical parameters at baseline according to calcium quintiles (n=4246).

Calcium (mmol/l)	Q1 (≤2.16 mmol/l)	Q2 (>2,16 - ≤2.24 mmol/l)	Q3 (>2.24 - ≤2.30 mmol/l)	Q4 (>2.30 - ≤2.37 mmol/l)	Q5 (>2.37 mmol/l)
N (%)	878 (20.7)	855 (20.1)	846 (19.9)	835 (19.7)	832 (19.6)
Demographics					
Age, years	60.2 ± 11.8	60.7 ± 11.8	60.6 ± 12.0	59.8 ± 11.8	60.1 ± 11.9
Male, N (%)	569 (13.4)	546 (12.9)	539 (12.7)	496 (11.7)	459 (10.8)
Laboratory Measures					
eGFR (ml/min per 1.73 m^2)	46.9 ± 17.6	46.9 ± 17.6	49.3 ± 17.6	48.1 ± 17.0	50.2 ± 17.5
UACR (mg/g)	88.3 (12.9-827.0)	54.9 (10.8-464.2)	40.4 (8.8-260.7)	48.2 (9.1-334.4)	44.7 (9.0-248.4)
hsCRP (mg/l)	2.6 (1.0-5.9)	2.3 (1.0-5.0)	2.2 (1.1-4.9)	2.1 (1.0-4.9)	2.2 (1.1-4.8)
Serum albumin (mg/l)	36.4 (33.3-38.8)	37.9 (35.8-39.9)	38.6 (36.6-40.7)	39.4 (37.3-41.4)	40.5 (38.7-42.6)
LDL cholesterol (mg/dl)	106.9 (82.4-137.5)	112.1 (87.9-140.5)	112.9 (88.3-141.8)	115.4 (92.5-142.2)	115.9 (91.0-148.0)
HDL cholesterol (mg/dl)	48.2 (37.3-59.1)	46.7 (38.2-58.2)	48.5 (39.3-60.4)	48.4 (38.9-61.0)	50.4 (41.1-63.4)
CVD risk factors					
Systolic BP (mmHg)	140.2 ± 21.5	139.4 ± 20.0	139.9 ± 21.1	138.6 ± 19.3	139.1 ± 19.9
Diastolic BP (mmHg)	78.3 ± 12.6	78.8 ± 12.1	78.8 ± 11.6	80.0 ± 11.3	79.6 ± 11.7
BMI (kg/m^2)	30.0 ± 6.0	30.1 ± 6.0	30.0 ± 6.1	29.7 ± 5.9	29.7 ± 6.0
Diabetes, N (%)	320 (7.5)	313 (7.4)	307 (7.2)	284 (6.7)	298 (7.0)
Previous CVD, N (%)	245 (5.8)	233 (5.5)	219 (5.2)	224 (5.3)	202 (4.8)
Smoking history					
never, N (%)	359 (8.5)	331 (7.8)	355 (8.4)	332 (7.8)	322 (7.6)
former, N (%)	382 (9.0)	371 (8.8)	347 (8.2)	359 (8.5)	376 (8.9)
current, N (%)	133 (3.1)	151 (3.6)	144 (3.4)	139 (3.3)	133 (3.1)
unknown, N	4 (0.1)	2 (0.0)	0 (0.0)	5 (0.1)	1 (0.0)
Relevant Medication					
RASi, N (%)	742 (17.5)	717 (16.9)	718 (16.9)	708 (16.7)	715 (16.8)
Statins, N (%)	435 (10.3)	412 (9.8)	395 (9.4)	416 (9.9)	401 (9.5)
Betablocker, N (%)	492 (11.7)	493 (11.7)	455 (10.8)	451 (10.7)	469 (11.1)
Aldosterone antagonists, N (%)	77 (1.8)	64 (1.5)	50 (1.2)	68 (1.6)	84 (2.0)
Thrombocyte aggregation inhibitors, N (%)	283 (6.7)	300 (7.1)	309 (7.3)	321 (7.6)	302 (7.2)
Vitamin D therapy, N (%)	307 (7.3)	257 (6.1)	240 (5.7)	252 (5.9)	264 (6.3)

Continuous variables are presented as mean and standard deviation or median and interquartile range. Categorical variables are presented as absolute numbers and as percentage of overall study population. eGFR, estimated glomerular filtration rate; UACR, urine albumin creatinine ratio; hsCRP, high-sensitivity C-reactive protein; LDL, low density lipoprotein; HDL, high density lipoprotein; BP, blood pressure; CVD, cardiovascular disease; BMI; body mass index, RASi; renin angiotensin system inhibitor.

Table S4. Demographics and clinical parameters at baseline according to 25-OH vitamin D quintiles (n=4246).

25-OH vitamin D (ng/ml)	Q1 (≤15.3 ng/ml)	Q2 (>15.3 - ≤20.5 ng/ml)	Q3 (>20.5 - ≤25.7 ng/ml)	Q4 (>25.7 - ≤31.7 ng/ml)	Q5 (>31.7 ng/ml)
N (%)	857 (20.2)	853 (20.1)	851 (20.0)	840 (19.8)	845 (19.9)
Demographics					
Age, years	60.4 ± 12.3	61.1 ± 11.3	60.6 ± 11.3	60.6 ± 11.6	58.6 ± 12.7
Male, N (%)	491 (11.6)	555 (13.1)	545 (12.8)	527 (12.4)	491 (11.6)
Laboratory Measures					
eGFR (ml/min per 1.73 m^2)	49.1 ± 19.0	57.7 ± 17.0	47.9 ± 15.9	48.3 ± 17.0	48.4 ± 18.4
UACR (mg/g)	76.4 (12.3-616.2)	46.1 (9.2-370.0)	42.8 (8.6-283.8)	51.0 (10.7-296.4)	59.5 (9.8-364.2)
hsCRP (mg/l)	2.6 (1.2-5.9)	2.5 (1.1-5.2)	2.3 (1.0-4.9)	2.2 (1.0-4.7)	2.1 (1.0-4.9)
Serum albumin (mg/l)	37.9 (35.2-40.4)	38.6 (36.3-409)	38.7 (36.6-40.7)	38.8 (34.5-41.1)	39.0 (36.7-41.0).
LDL cholesterol (mg/dl)	115.2 (87.7-146.5)	110.6 (86.8-138.7)	111.1 (87.6-141.9)	112.2 (89.5-138.3)	114.1 (88.8-145.7)
HDL cholesterol (mg/dl)	47.0 (38.9-58.8)	45.8 (37.4-59.5)	48.2 (39.4-60.7)	47.8 (39.5-59.5)	50.7 (40.3-65.3)
CVD risk factors					
Systolic BP (mmHg)	140.9 ± 20.8	140.3 ± 20.5	139.7 ± 20.7	139.6 ± 20.6	139.6 ± 19.0
Diastolic BP (mmHg)	78.6 ± 12.5	79.1 ± 12.0	79.4 ± 11.7	79.2 ± 11.9	79.2 ± 11.3
BMI (kg/m^2)	30.7 ± 6.1	30.6 ± 6.5	29.9 ± 6.0	29.8 ± 5.6	28.4 ± 5.4
Diabetes, N (%)	376 (8.9)	344 (8.1)	327 (7.7)	259 (6.1)	216 (5.1)
Previous CVD, N (%)	257 (6.1)	235 (5.5)	228 (5.4)	199 (4.7)	204 (4.8)
Smoking history					
never, N (%)	321 (37.5)	339 (39.7)	344 (40.4)	345 (41.1)	350 (41.4)
former, N (%)	346 (40.4)	377 (44.2)	382 (44.9)	375 (44.6)	355 (42.0)
current, N (%)	188 (21.9)	135 (15.8)	124 (14.6)	118 (14.0)	135 (16.0)
unknown, N	2 (0.0)	2 (0.0)	1 (0.0)	2 (0.0)	5 (0.1)
Relevant Medication					
RASi, N (%)	728 (17.1)	718 (16.9)	737 (17.4)	712 (16.8)	705 (16.6)
Statins, N (%)	417 (9.9)	422 (10.0)	410 (9.7)	403 (9.6)	407 (9.7)
Betablocker, N (%)	489 (11.6)	490 (11.6)	466 (11.1)	464 (11.0)	451 (10.7)
Aldosterone antagonists, N (%)	81 (1.9)	71 (1.7)	61 (1.4)	66 (1.6)	64 (1.5)
Thrombocyte aggregation inhibitors, N (%)	334 (7.9)	326 (7.7)	316 (7.5)	274 (6.5)	265 (6.3)
Vitamin D therapy, N (%)	260 (6.2)	239 (5.7)	255 (6.1)	272 (6.5)	294 (7.0)

Continuous variables are presented as mean and standard deviation or median and interquartile range. Categorical variables are presented as absolute numbers and as percentage of overall study population. eGFR, estimated glomerular filtration rate; UACR, urine albumin creatinine ratio; hsCRP, high-sensitivity C-reactive protein; LDL, low density lipoprotein; HDL, high density lipoprotein; BP, blood pressure; CVD, cardiovascular disease; BMI; body mass index, RASi; renin angiotensin system inhibitor.

Table S5. Demographics and clinical parameters at baseline according to bone alkaline phosphatase (BAP) quintiles (n=4246).

BAP (µg/l)	Q1 (≤12.3 µg/l)	Q2 (<12.3 - ≤15.1 µg/l)	Q3 (<15.1 - ≤18.2 µg/l)	Q4 (<18.2 - ≤22.8 µg/l)	Q5 (>22.8 µg/l)
N (%)	859 (20.2)	840 (19.8)	872 (20.5)	829 (19.5)	846 (19.9)
Demographics					
Age, years	58.7 ± 12.7	59.6 ± 12.2	60.8 ± 11.5	60.5 ± 11.9	61.8 ± 10.9
Male, N (%)	563 (13.3)	559 (13.2)	546 (12.9)	509 (12.0)	432 (10.2)
Laboratory Measures					
eGFR (ml/min per 1.73 m^2)	49.7 ± 18.6	50.2 ± 18.4	49.2 ± 17.2	47.1 ± 16.9	45.2 ± 15.9
UACR (mg/g)	56.4 (9.9-420.6)	55.7 (10.3-403.5)	44.4 (8.9-338.3)	54.2 (12.3-388.8)	52.1 (9.6-344.6)
hsCRP (mg/l)	1.8 (0.8-3.9)	2.1 (1.0-4.5)	2.4 (1.1-5.2)	2.6 (1.2-5.5)	3.1 (1.5-6.9)
Serum albumin (mg/l)	38.4 (35.7-40.8)	38.7 (36.4-40.9)	39.0 (36.3-40.7)	38.7 (36.2-41.0)	38.3 (36.2-40.8)
LDL cholesterol (mg/dl)	113.5 (91.3-142.6)	113.1 (87.7-141.6)	113.5 (87.4-141.9)	111.8 (86.7-140.5)	111.5 (86.8-143.8)
HDL cholesterol (mg/dl)	50.3 (39.8-64.6)	48.4 (39.8-61.5)	47.1 (39.4-58.3)	47.1 (39.5-59.9)	47.0 (38.0-60.1)
CVD risk factors					
Systolic BP (mmHg)	138.4 ± 20.5	138.0 ± 19.4	139.3 ± 19.8	140.1 ± 20.6	141.5 ± 21.3
Diastolic BP (mmHg)	79.1 ± 11.7	78.7 ± 11.7	79.0 ± 11.4	78.8 ± 12.6	79.8 ± 12.1
BMI (kg/m^2)	28.7 ± 5.4	29.5 ± 5.7	30.1 ± 6.1	30.3 ± 5.9	30.8 ± 6.5
Diabetes, N (%)	266 (6.3)	261 (6.1)	311 (7.3)	319 (7.5)	365 (8.6)
Previous CVD, N (%)	203 (4.8)	212 (5)	243 (5.7)	228 (5.4)	237 (5.6)
Smoking history					
Never, N (%)	342 (8.1)	320 (7.6)	354 (8.4)	325 (7.7)	358 (8.5)
Former, N (%)	378 (8.9)	376 (8.9)	381 (9.0)	369 (8.7)	331 (7.8)
Current, N (%)	137 (3.2)	141 (3.3)	135 (3.2)	134 (3.2)	153 (3.6)
Unknown, N	2 (0.0)	3 (0.0)	2 (0.0)	1 (0.0)	4 (0.1)
Relevant Medication					
RASi, N (%)	754 (17.8)	721 (17.0)	722 (17.0)	716 (16.9)	687 (16.2)
Statins, N (%)	415 (9.8)	421 (10.0)	445 (10.6)	378 (9.0)	400 (9.5)
Betablocker, N (%)	468 (11.1)	461 (10.9)	473 (11.2)	485 (11.5)	473 (11.2)
Aldosterone antagonists, N (%)	69 (1.6)	50 (1.2)	103 (2.4)	69 (1.6)	52 (1.2)
Thrombocyte aggregation inhibitors, N (%)	276 (6.5)	292 (6.9)	314 (7.4)	325 (7.7)	308 (7.3)
Vitamin D therapy, N (%)	306 (7.3)	260 (6.2)	250 (5.9)	242 (5.7)	262 (6.2)

Continuous variables are presented as mean and standard deviation or median and interquartile range. Categorical variables are presented as absolute numbers and as percentage of overall study population. BAP, bone alkaline phosphatase; eGFR, estimated glomerular filtration rate; UACR, urine albumin creatinine ratio; hsCRP, high-sensitivity C-reactive protein; LDL, low density lipoprotein; HDL, high density lipoprotein; BP, blood pressure; CVD, cardiovascular disease; BMI; body mass index, RASi; renin angiotensin system inhibitor.

Table S6. Demographics and clinical parameters at baseline according to C telopeptide of type 1 collagen (CTX1) quintiles (n=4246).

CTX1 (ng/ml)	Q1 (≤0.1 ng/ml)	Q2 (>0.1 - ≤0.2 ng/ml)	Q3 (>0.2 - ≤0.3 ng/ml)	Q4 (>0.3 - ≤0.5 ng/ml)	Q5 (>0.5 ng/ml)
N (%)	852 (20.0)	856 (20.2)	842 (19.8)	850 (20.0)	849 (19.9)
Demographics					
Age, years	60.4 ± 11.7	60.7 ± 11.4	61.2 ± 11.3	60.7 ± 11.8	58.5 ± 12.9
Male, N (%)	544 (12.8)	538 (12.7)	525 (12.4)	507 (11.9)	495 (11.7)
Laboratory Measures					
eGFR (ml/min per 1.73 m^2)	56.5 ± 19.6	51.9 ± 16.3	48.4 ± 16.2	44.6 ± 15.4	39.8 ± 14.8
UACR (mg/g)	39.9 (8.3-356.8)	38.3 (8.2-251.3)	45.5 (9.2-292.8)	63.5 (10.6-402.9)	105.6 (15.0-639.4)
hsCRP (mg/l)	2.3 (1.1-4.9)	2.4 (1.1-5.1)	2.3 (1.1-5.0)	2.4 (1.1-5.2)	2.2 (0.9-5.4)
Serum albumin (mg/l)	39.0 (36.2-41.1)	38.8 (36.6-40.8)	38.9 (36.7-40.8)	38.5 (36.0-40.7)	38.0 (35.8-40.6)
LDL cholesterol (mg/dl)	112.2 (87.1-139.9)	115.5 (90.8-147.7)	112.0 (86.6-140.4)	115.1 (92.2-140.9)	108.6 (84.3-142.5)
HDL cholesterol (mg/dl)	47.8 (38.5-60.7)	47.7 (38.9-58.9)	47.2 (39.2-60.1)	48.7 (39.5-62.1)	48.4 (38.7-61.8)
CVD risk factors					
Systolic BP (mmHg)	139.8 ± 19.6	140.3 ± 20.6	139.9 ± 21.2	139.1 ± 20.0	138.1 ± 20.4
Diastolic BP (mmHg)	79.6 ± 11.3	79.7 ± 11.5	78.5 ± 12.1	78.4 ± 12.0	79.1 ± 12.5
BMI (kg/m^2)	30.2 ± 6.0	30.3 ± 5.8	30.2 ± 6.1	29.6 ± 5.7	29.0 ± 6.3
Diabetes, N (%)	339 (8.0)	307 (7.2)	310 (7.3)	291 (6.9)	275 (6.5)
Previous CVD, N (%)	229 (5.4)	228 (5.4)	235 (5.5)	208 (4.9)	223 (5.3)
Smoking history					
never, N (%)	358 (8.5)	326 (7.7)	334 (7.9)	336 (7.9)	345 (8.1)
former, N (%)	365 (8.6)	388 (9.2)	374 (8.8)	375 (8.9)	333 (7.9)
current, N (%)	125 (3.0)	139 (3.3)	133 (3.1)	138 (3.3)	165 (3.9)
unknown, N	4 (0.1)	3 (0.0)	1 (0.0)	1 (0.0)	3 (0.0)
Relevant Medication					
RASi, N (%)	728 (17.1)	717 (16.9)	723 (17.0)	718 (16.9)	714 (16.8)
Statins, N (%)	432 (10.2)	419 (9.9)	405 (9.6)	409 (9.7)	394 (9.3)
Betablocker, N (%)	461 (10.9)	481 (11.4)	481 (11.5)	453 (10.7)	482 (11.4)
Aldosterone antagonists, N (%)	69 (1.6)	83 (2.0)	69 (1.6)	62 (1.5)	67 (1.6)
Thrombocyte aggregation inhibitors, N (%)	289 (6.9)	300 (7.1)	326 (7.7)	302 (7.2)	298 (7.1)
Vitamin D therapy, N (%)	246 (5.8)	243 (5.8)	249 (5.9)	265 (6.3)	317 (7.5)

Continuous variables are presented as mean and standard deviation or median and interquartile range. Categorical variables are presented as absolute numbers and as percentage of overall study population. CTX1, C-Telopeptide of Type I Collagen; eGFR, estimated glomerular filtration rate; UACR, urine albumin creatinine ratio; hsCRP, high-sensitivity C-reactive protein; LDL, low density lipoprotein; HDL, high density lipoprotein; BP, blood pressure; CVD, cardiovascular disease; BMI; body mass index, RASi; renin angiotensin system inhibitor.

Table S7: Demographics and clinical parameters at baseline according to phosphate quintiles (n=4246).

Phosphate (mmol/l)	Q1 (≤0.94 mmol/l)	Q2 (>0.94 - ≤1.06 mmol/l)	Q3 (>1.06 - ≤1.16 mmol/l)	Q4 (>1.16 - ≤1.27 mmol/l)	Q5 (>1.27 mmol/l)
N (%)	871 (20.5)	882 (20.8)	872 (20.5)	824 (19.4)	797 (18.8)
Demographics					
Age, years	61.0 ± 11.3	60.8 ± 11.5	60.8 ± 11.4	60.2 ± 11.9	58.4 ± 13.2
Male, N (%)	686 (16.2)	602 (14.2)	509 (12)	433 (10.2)	379 (8.9)
Laboratory Measures					
eGFR (ml/min per 1.73 m^2)	50.6 ± 15.9	49.2 ± 16.2	48.6 ± 17.1	47.7 ± 17.9	45.0 ± 20.0
UACR (mg/g)	38.7 (8.2-249.7)	51.1 (9.2-313.9)	44.5 (9.0-356.0)	55.0 (11.4-528.2)	87.5 (11.9-700.1)
hsCRP (mg/l)	2.5 (1.1-5.8)	2.5 (1.2-5.6)	2.2 (1.0-5.4)	2.2 (1.0-4.5)	2.3 (0.9-4.7)
Serum albumin (mg/l)	38.7 (36.6-40.8)	38.5 (36.1-40.9)	38.8 (36.4-40.8)	38.8 (36.3-40.8)	38.5 (35.4-41.0)
LDL cholesterol (mg/dl)	111.6 (86.8-140.1)	111.3 (86.8-139.8)	116.4 (90.1-142.5)	112.0 (89.2-144.6)	111.9 (87.8-147.0)
HDL cholesterol (mg/dl)	45.5 (38.2-55.7)	45.3 (38.0-56.9)	49.7 (40.2-62.4)	49.9 (40.1-62.1)	50.8 (39.9-66.1)
CVD risk factors					
Systolic BP (mmHg)	140.6 ± 19.6	139.4 ± 20.5	140.0 ± 20.7	139.5 ± 20.3	137.5 ± 20.8
Diastolic BP (mmHg)	80.2 ± 12.0	79.2 ± 11.8	79.6 ± 11.7	78.6 ± 11.2	77.8 ± 12.7
BMI (kg/m^2)	30.3 ± 5.7	30.2 ± 6.0	29.8 ± 6.0	29.7 ± 5.9	29.4 ± 6.3
Diabetes, N (%)	305 (7.2)	323 (7.6)	288 (6.8)	287 (6.8)	319 (7.5)
Previous CVD, N (%)	234 (5.5)	236 (5.6)	219 (5.2)	219 (5.2)	215 (5.1)
Smoking history					
never, N (%)	327 (7.7)	341 (8.1)	372 (8.8)	324 (7.7)	335 (7.9)
former, N (%)	419 (9.9)	398 (9.4)	378 (8.9)	340 (8.0)	300 (7.1)
current, N (%)	124 (2.9)	140 (3.3)	118 (2.8)	159 (3.8)	159 (3.8)
unknown, N	1 (0.0)	3 (0.0)	4 (0.1)	1 (0.0)	3 (0.0)
Relevant Medication					
RASi, N (%)	732 (17.2)	745 (17.5)	737 (17.4)	709 (16.7)	677 (15.9)
Statins, N (%)	432 (10.2)	430 (10.2)	404 (9.6)	388 (9.2)	405 (9.6)
Betablocker, N (%)	451 (10.7)	506 (12.0)	458 (10.9)	459 (10.9)	486 (11.5)
Aldosterone antagonists, N (%)	74 (1.8)	75 (1.8)	73. (1.7)	59 (1.4)	63 (1.5)
Thrombocyte aggregation inhibitors, N (%)	301 (7.1)	298 (7.1)	306 (7.3)	290 (6.9)	320 (7.6)
Vitamin D therapy, N (%)	273 (6.5)	270 (6.4)	258 (6.1)	253 (6.0)	266 (6.3)

Continuous variables are presented as mean and standard deviation or median and interquartile range. Categorical variables are presented as absolute numbers and as percentage of overall study population. eGFR, estimated glomerular filtration rate; UACR, urine albumin creatinine ratio; hsCRP, high-sensitivity C-reactive protein; LDL, low density lipoprotein; HDL, high density lipoprotein; BP, blood pressure; CVD, cardiovascular disease; BMI; body mass index, RASi; renin angiotensin system inhibitor.

Table S8: Demographics and clinical parameters at baseline according to procollagen 1 intact N terminal (P1NP) quintiles (n=4246).

P1NP (ng/ml)	Q1 (≤32.2 ng/ml)	Q2 (>32.2 - ≤42.0 ng/ml)	Q3 (>42.0 - ≤52.9 ng/ml)	Q4 (>52.9 - ≤70.1 ng/ml)	Q5 (>70.1 ng/ml)
N (%)	850 (20.0)	853 (20.1)	845 (19.9)	851 (20.0)	847 (20.0)
Demographics					
Age, years	60.7 ± 11.2	61.3 ± 10.7	60.7 ± 11.6	60.2 ± 12.1	58.5 ± 13.4
Male, N (%)	605 (14.2)	589 (13.9)	549 (12.9)	479 (11.3)	387 (9.1)
Laboratory Measures					
eGFR (ml/min per 1.73 m^2)	49.8 ± 17.7	48.4 ± 16.7	48.5 ± 17.5	48.2 ± 17.3	46.4 ± 18.1
UACR (mg/g)	47.8 (10.4-384.9)	58.3 (9.3-449.5)	44.8 (8.4-337.9)	53.2 (11.0-368.0)	57.5 (10.3-394.2)
hsCRP (mg/l)	2.3 (1.0-5.1)	2.5 (1.1-5.5)	2.2 (1.0-4.6)	2.3 (1.0-5.3)	2.3 (1.0-5.2)
Serum albumin (mg/l)	38.5 (35.7-40.7)	38.6 (36.2-40.7)	39.0 (36.5-41.2)	38.8 (36.6 (40.8)	38.3 (36.0-41.1)
LDL cholesterol (mg/dl)	110.8 (86.3-142.6)	113.6 (89.4-141.2)	114.6 (88.2-141.1)	111.9 (89.2-141.3)	112.8 (88.1-144.2)
HDL cholesterol (mg/dl)	46.9 (37.8-59.5)	46.0 (37.8-58.2)	47.3 (39.2-59.1)	49.2 (40.8-61.8)	50.5 (40.7-64.3)
CVD risk factors					
Systolic BP (mmHg)	139.1 ± 20.5	140.3 ± 20.1	139.8 ± 20.6	140.3 ± 20.7	137.6 ± 19.9
Diastolic BP (mmHg)	78.8 ± 11.9	79.1 ± 11.6	79.4 ± 12.4	79.8 ± 11.7	78.3 ± 11.9
BMI (kg/m^2)	30.0 ± 5.6	30.6 ± 6.1	29.9 ± 5.8	29.7 ± 5.9	29.2 ± 6.5
Diabetes, N (%)	355 (8.4)	350 (8.2)	280 (6.6)	265 (6.2)	272 (6.4)
Previous CVD, N (%)	234 (5.5)	259 (6.1)	228 (5.4)	207 (4.9)	195 (4.6)
Smoking history					
never, N (%)	309 (7.3)	313 (7.4)	345 (8.1)	378 (8.9)	354 (8.4)
former, N (%)	402 (9.5)	404 (9.5)	368 (8.7)	331 (7.8)	330 (7.8)
current, N (%)	136 (3.2)	133 (3.1)	131 (3.1)	141 (3.3)	159 (3.8)
unknown, N	3 (0.0)	3 (0.0)	1 (0.0)	1 (0.0)	4 (0.1)
Relevant Medication					
RASi, N (%)	746 (17.6)	744 (17.5)	719 (16.9)	718 (16.9)	673 (15.9)
Statins, N (%)	446 (10.6)	435 (10.3)	407 (9.7)	395 (9.4)	376 (8.9)
Betablocker, N (%)	508 (12.0)	506 (12.0)	456 (10.8)	455 (10.8)	435 (10.3)
Aldosterone antagonists, N (%)	101 (2.4)	76 (1.8)	58 (1.4)	47 (1.1)	61 (1.4)
Thrombocyte aggregation inhibitors, N (%)	303 (7.2)	338 (8.0)	297 (7.0)	309 (7.3)	268 (6.4)
Vitamin D therapy, N (%)	311 (7.4)	258 (6.1)	242 (5.7)	232 (5.5)	277 (6.6)

Continuous variables are presented as mean and standard deviation or median and interquartile range. Categorical variables are presented as absolute numbers and as percentage of overall study population. P1NP, Procollagen I Intact N-Terminal; eGFR, estimated glomerular filtration rate; UACR, urine albumin creatinine ratio; hsCRP, high-sensitivity C-reactive protein; LDL, low density lipoprotein; HDL, high density lipoprotein; BP, blood pressure; CVD, cardiovascular disease; BMI; body mass index, RASi; renin angiotensin system inhibitor.

Table S9. Association of fibroblast growth factor 23 (FGF23) with different outcomes.

FGF23		model 1 (univariate)	model 2	model 3
		HR [95% CI]		
**non-CV death** 387/4245	HR per SD increase	1.05 [1.04; 1.06]	1.02 [1.01; 1.04]	1.02 [1.00; 1.03]
	Q1	1 (ref.)	1 (ref.)	1 (ref.)
	Q2	0.99 [0.63; 1.55]	1.00 [0.63; 1.60]	1.06 [0.66; 1.70]
	Q3	1.78 [1.20; 2.66]	1.51 [0.99; 2.29]	1.46 [0.95; 2.22]
	Q4	2.53 [1.73; 3.69]	1.85 [1.24; 2.77]	1.78 [1.18; 2.67]
	Q5	4.80 [3.37; 6.85]	2.80 [1.90; 4.12]	2.50 [1.68; 3.73]
**CV death** 173/4245	HR per SD increase	1.1 [0.95; 1.28]	1.03 [1.02; 1.05]	1.02 [1.00; 1.04]
	Q1	1 (ref.)	1 (ref.)	1 (ref.)
	Q2	0.80 [0.42; 1.52]	0.72 [0.37; 1.41]	0.68 [0.35; 1.34]
	Q3	1.08 [0.59; 1.96]	0.79 [0.43; 1.46]	0.75 [0.40; 1.40]
	Q4	2.01 [1.18; 3.42]	1.22 [0.71; 2.11]	1.15 [0.66; 2.02]
	Q5	4.14 [2.55; 6.73]	1.87 [1.11; 3.13]	1.73 [1.01; 2.97]
**MACE** 645/4244	HR per SD increase	1.04 [1.03; 1.05]	1.03 [1.02; 1.04]	1.02 [1.01; 1.03]
	Q1	1 (ref.)	1 (ref.)	1 (ref.)
	Q2	0.87 [0.65; 1.16]	0.81 [0.60; 1.10]	0.79 [0.59; 1.08]
	Q3	1.22 [0.94; 1.60]	1.05 [0.80; 1.38]	1.06 [0.80; 1.40]
	Q4	1.76 [1.37; 2.26]	1.23 [0.95; 1.61]	1.19 [0.91; 1.56]
	Q5	2.23 [1.74; 2.84]	1.36 [1.04; 1.77]	1.26 [0.95; 1.67]
**CHF** 368/4245	HR per SD increase	1.06 [1.05; 1.07]	1.04 [1.02; 1.05]	1.03 [1.01; 1.05]
	Q1	1 (ref.)	1 (ref.)	1 (ref.)
	Q2	0.86 [0.56; 1.32]	0.82 [0.52; 1.27]	0.75 [0.48; 1.17]
	Q3	1.32 [0.89; 1.96]	1.05 [0.70; 1.57]	0.97 [0.65; 1.46]
	Q4	2.12 [1.47; 3.04]	1.38 [0.95; 2.00]	1.20 [0.82; 1.76]
	Q5	3.96 [2.83; 5.55]	2.15 [1.50; 3.07]	1.77 [1.21; 2.57]

Results are presented as hazard ratios with 95%-confidence intervals given in parentheses.
Model 2: adjusted for age, sex, BMI, systolic blood pressure, LDL cholesterol, CRP, serum albumin, eGFR, UACR, diabetes mellitus, CVD, smoking, use of statins, use of RASi, use of thrombocyte aggregation inhibitors, use of betablockers, ongoing vitamin D therapy, and use of aldosterone antagonists.
Model 3: adjusted for parameters as in Model 2 plus OPG, BAP, calcium, phosphate, P1NP, CTX1, 25-OH vitamin D, and iPTH
Abbreviations: OPG, osteoprotegerin; HR, hazard ratio; SD, standard deviation; CV, cardiovascular; MACE, major adverse cardiac event; CHF, hospitalization due to congestive heart failure; eGFR, estimated glomerular filtration rate; UACR, urine albumin creatinine ratio; hsCRP, high-sensitivity C-reactive protein; LDL, low density lipoprotein; HDL, high density lipoprotein; BP, blood pressure; CVD, cardiovascular disease; BMI, body mass index; RASi, renin angiotensin system inhibitor; BAP, bone alkaline phosphatase; P1NP, procollagen 1 intact N-terminal propeptide; CTX1, C-telopeptide of Type 1 collagen; FGF23, fibroblast growth factor 23; iPTH, intact parathormone.

Table S10. Association of intact parathormone (iPTH) with different outcomes.

iPTH		model 1 (univariate)	model 2	model 3
		HR [95% CI]		
**non-CV death** 387/4245	HR per SD increase	1.24 [1.19; 1.30]	1.18 [1.11; 1.26]	1.12 [1.04; 1.22]
	Q1	1 (ref.)	1 (ref.)	1 (ref.)
	Q2	1.45 [0.96; 2.19]	1.10 [0.71; 1.72]	1.10 [0.70; 1.72]
	Q3	1.55 [1.03; 2.33]	1.11 [0.71; 1.72]	1.04 [0.66; 1.64]
	Q4	2.95 [2.04; 4.27]	1.93 [1.28; 2.90]	1.67 [1.09; 2.57]
	Q5	3.66 [2.55; 5.26]	2.22 [1.47; 3.35]	1.64 [1.04; 2.59]
**CV death** 173/4245	HR per SD increase	1.24 [1.16; 1.33]	1.11 [0.99; 1.25]	1.06 [0.92; 1.22]
	Q1	1 (ref.)	1 (ref.)	1 (ref.)
	Q2	0.94 [0.52; 1.71]	0.62 [0.34; 1.15]	0.61 [0.33; 1.13]
	Q3	1.30 [0.74; 2.26]	0.68 [0.38; 1.22]	0.65 [0.35; 1.19]
	Q4	2.04 [1.22; 3.41]	0.88 [0.51; 1.53]	0.74 [0.41; 1.34]
	Q5	2.95 [1.81; 4.81]	1.03 [0.60; 1.77]	0.69 [0.37; 1.28]
**MACE** 645/4244	HR per SD increase	1.21 [1.16; 1.27]	1.11 [1.04; 1.18]	1.07 [0.99; 1.15]
	Q1	1 (ref.)	1 (ref.)	1 (ref.)
	Q2	1.18 [0.89; 1.56]	0.96 [0.71; 1.28]	0.96 [0.71; 1.29]
	Q3	1.44 [1.09; 1.88]	1.00 [0.75; 1.34]	0.95 [0.71; 1.28]
	Q4	1.53 [1.17; 2.00]	0.92 [0.69; 1.24]	0.81 [0.59; 1.10]
	Q5	2.46 [1.91; 3.16]	1.36 [1.03; 1.80]	1.09 [0.79; 1.49]
**CHF** 368/4245	HR per SD increase	1.24 [1.19; 1.30]	1.16 [1.09; 1.24]	1.15 [1.06; 1.24]
	Q1	1 (ref.)	1 (ref.)	1 (ref.)
	Q2	1.11 [0.75; 1.66]	0.74 [0.48; 1.12]	0.72 [0.47; 1.09]
	Q3	1.41 [0.96; 2.06]	0.79 [0.52; 1.19]	0.76 [0.50; 1.15]
	Q4	2.16 [1.51; 3.08]	1.11 [0.76; 1.63]	0.99 [0.66; 1.50]
	Q5	3.05 [2.17; 4.30]	1.38 [0.94; 2.02]	1.09 [0.70; 1.67]

Results are presented as hazard ratios with 95%-confidence intervals given in parentheses.
Model 2: adjusted for age, sex, BMI, systolic blood pressure, LDL cholesterol, CRP, serum albumin, eGFR, UACR, diabetes mellitus, CVD, smoking, use of statins, use of RASi, use of thrombocyte aggregation inhibitors, use of betablockers, ongoing vitamin D therapy, and use of aldosterone antagonists.
Model 3: adjusted for parameters as in Model 2 plus OPG, BAP, calcium, phosphate, P1NP, CTX1, 25-OH vitamin D, and FGF23
Abbreviations: OPG, osteoprotegerin; HR, hazard ratio; SD, standard deviation; CV, cardiovascular; MACE, major adverse cardiac event; CHF, hospitalization due to congestive heart failure; eGFR, estimated glomerular filtration rate; UACR, urine albumin creatinine ratio; hsCRP, high-sensitivity C-reactive protein; LDL, low density lipoprotein; HDL, high density lipoprotein; BP, blood pressure; CVD, cardiovascular disease; BMI, body mass index; RASi, renin angiotensin system inhibitor; BAP, bone alkaline phosphatase; P1NP, procollagen 1 intact N-terminal propeptide; CTX1, C-telopeptide of type 1 collagen; FGF23, fibroblast growth factor 23; iPTH, intact parathormone.

Table S11. Association of serum calcium levels with different outcomes.

Calcium		model 1 (univariate)	model 2	model 3
		HR [95% CI]		
**non-CV death** 387/4245	HR per SD increase	0.01 [0.00; 0.06]	0.30 [0.04; 2.02]	0.57 [0.08; 4.04]
	Q1	1 (ref.)	1 (ref.)	1 (ref.)
	Q2	0.68 [0.51; 0.92]	0.86 [0.63; 1.17]	0.92 [0.67; 1.27]
	Q3	0.69 [0.51; 0.92]	0.94 [0.68; 1.30]	1.09 [0.78; 1.52]
	Q4	0.57 [0.42; 0.78]	0.92 [0.65; 1.29]	1.05 [0.74; 1.48]
	Q5	0.57 [0.42; 0.78]	0.94 [0.66; 1.34]	1.11 [0.77; 1.61]
**CV death** 173/4245	HR per SD increase	0.07 [0.01; 0.77]	1.87 [0.12; 29.96]	2.55 [0.15; 44.48]
	Q1	1 (ref.)	1 (ref.)	1 (ref.)
	Q2	0.96 [0.63; 1.47]	1.19 [0.76; 1.88]	1.23 [0.78; 1.95]
	Q3	0.66 [0.41; 1.06]	1.06 [0.65; 1.74]	1.23 [0.74; 2.06]
	Q4	0.59 [0.36; 0.95]	1.03 [0.61; 1.74]	1.07 [0.62; 1.82]
	Q5	0.70 [0.44; 1.11]	1.18 [0.70; 1.99]	1.33 [0.76; 2.30]
**MACE** 645/4244	HR per SD increase	0.17 [0.05; 0.62]	1.06 [0.24; 4.64]	1.48 [0.33; 6.58]
	Q1	1 (ref.)	1 (ref.)	1 (ref.)
	Q2	1.00 [0.80; 1.26]	1.11 [0.87; 1.42]	1.16 [0.91; 1.48]
	Q3	0.89 [0.70; 1.13]	1.16 [0.90; 1.50]	1.27 [0.98; 1.65]
	Q4	0.82 [0.64; 1.05]	1.07 [0.82; 1.39]	1.13 [0.86; 1.49]
	Q5	0.72 [0.56; 0.92]	0.93 [0.70; 1.24]	0.99 [0.73; 1.33]
**CHF** 368/4245	HR per SD increase	1.00 [1.00; 1.01]	0.20 [0.03; 1.38]	0.33 [0.05; 2.38]
	Q1	1 (ref.)	1 (ref.)	1 (ref.)
	Q2	0.79 [0.59; 1.07]	0.86 [0.63; 1.18]	0.89 [0.65; 1.23]
	Q3	0.62 [0.45; 0.85]	0.79 [0.57; 1.12]	0.90 [0.63; 1.27]
	Q4	0.73 [0.54; 0.99]	0.99 [0.71; 1.39]	1.08 [0.77; 1.52]
	Q5	0.66 [0.48; 0.91]	0.85 [0.60; 1.22]	0.91 [0.63; 1.32]

Results are presented as hazard ratios with 95%-confidence intervals given in parentheses.
Model 2: adjusted for age, sex, BMI, systolic blood pressure, LDL cholesterol, CRP, serum albumin, eGFR, UACR, diabetes mellitus, CVD, smoking, use of statins, use of RASi, use of thrombocyte aggregation inhibitors, use of betablockers, ongoing vitamin D therapy, and use of aldosterone antagonists.
Model 3: adjusted for parameters as in Model 2 plus OPG, BAP, phosphate, P1NP, CTX1, 25-OH vitamin D, FGF23, and iPTH.
Abbreviations: OPG, osteoprotegerin; HR, hazard ratio; SD, standard deviation; CV, cardiovascular; MACE, major adverse cardiac event; CHF, hospitalization due to congestive heart failure; eGFR, estimated glomerular filtration rate; UACR, urine albumin creatinine ratio; hsCRP, high-sensitivity C-reactive protein; LDL, low density lipoprotein; HDL, high density lipoprotein; BP, blood pressure; CVD, cardiovascular disease; BMI, body mass index; RASi, renin angiotensin system inhibitor; BAP, bone alkaline phosphatase; P1NP, procollagen 1 intact N-terminal propeptide; CTX1, C-telopeptide of type 1 collagen; iFGF23, intact fibroblast growth factor 23; iPTH, intact parathormone.

Table S12. Association of 25-OH vitamin D with different outcomes.

25-OH vitamin D		model 1 (univariate)	model 2	model 3
		HR [95% CI]		
**non-CV death** 387/4245	HR per SD increase	0.98 [0.97; 0.99]	0.98 [0.97; 1.00]	0.99 [0.98; 1.00]
	Q1	1 (ref.)	1 (ref.)	1 (ref.)
	Q2	0.66 [0.49; 0.89]	0.65 [0.47; 0.88]	0.68 [0.50; 0.94]
	Q3	0.62 [0.46; 0.84]	0.61 [0.45; 0.84]	0.71 [0.51; 0.98]
	Q4	0.65 [0.49; 0.88]	0.69 [0.50; 0.95]	0.82 [0.59; 1.14]
	Q5	0.56 [0.41; 0.76]	0.58 [0.41; 0.82]	0.70 [0.49; 1.01]
**CV death** 173/4245	HR per SD increase	0.96 [0.94; 0.98]	0.97 [0.95; 0.99]	0.98 [0.96; 1.00]
	Q1	1 (ref.)	1 (ref.)	1 (ref.)
	Q2	0.85 [0.56; 1.30]	0.80 [0.51; 1.24]	0.78 [0.50; 1.23]
	Q3	0.94 [0.62; 1.42]	1.01 [0.66; 1.53]	1.05 [0.68; 1.62]
	Q4	0.53 [0.33; 0.86]	0.56 [0.33; 0.95]	0.59 [0.35; 1.02]
	Q5	0.38 [0.22; 0.65]	0.49 [0.27; 0.86]	0.47 [0.26; 0.85]
**MACE** 645/4244	HR per SD increase	0.97 [0.96; 0.98]	0.98 [0.97; 0.99]	0.99 [0.98; 1.00]
	Q1	1 (ref.)	1 (ref.)	1 (ref.)
	Q2	0.88 [0.70; 1.10]	0.92 [0.73; 1.16]	0.95 [0.75; 1.20]
	Q3	0.88 [0.70; 1.09]	1.01 [0.80; 1.27]	1.05 [0.83; 1.33]
	Q4	0.54 [0.42; 0.70]	0.68 [0.52; 0.90]	0.72 [0.55; 0.96]
	Q5	0.51 [0.40; 0.67]	0.65 [0.49; 0.86]	0.67 [0.50; 0.90]
**CHF** 368/4245	HR per SD increase	0.97 [0.96; 0.98]	0.98 [0.97; 1.00]	0.99 [0.98; 1.01]
	Q1	1 (ref.)	1 (ref.)	1 (ref.)
	Q2	0.84 [0.63; 1.12]	0.87 [0.65; 1.18]	0.90 [0.66; 1.22]
	Q3	0.66 [0.49; 0.90]	0.76 [0.55; 1.04]	0.80 [0.57; 1.11]
	Q4	0.59 [0.43; 0.81]	0.73 [0.52; 1.03]	0.81 [0.57; 1.15]
	Q5	0.51 [0.37; 0.72]	0.73 [0.51; 1.05]	0.79 [0.54; 1.14]

Results are presented as hazard ratios with 95%-confidence intervals given in parentheses.
Model 2: adjusted for age, sex, BMI, systolic blood pressure, LDL cholesterol, CRP, serum albumin, eGFR, UACR, diabetes mellitus, CVD, smoking, use of statins, use of RASi, use of thrombocyte aggregation inhibitors, use of betablockers, ongoing vitamin D therapy, and use of aldosterone antagonists.
Model 3: adjusted for parameters as in Model 2 plus OPG, BAP, calcium, phosphate, P1NP, CTX1, FGF23, and iPTH.
Abbreviations: OPG, osteoprotegerin; HR, hazard ratio; SD, standard deviation; CV, cardiovascular; MACE, major adverse cardiac event; CHF, hospitalization due to congestive heart failure; eGFR, estimated glomerular filtration rate; UACR, urine albumin creatinine ratio; hsCRP, high-sensitivity C-reactive protein; LDL, low density lipoprotein; HDL, high density lipoprotein; BP, blood pressure; CVD, cardiovascular disease; BMI, body mass index; RASi, renin angiotensin system inhibitor; BAP, bone alkaline phosphatase; P1NP, procollagen 1 intact N-terminal propeptide; CTX1, C-telopeptide of type 1 collagen; FGF23, fibroblast growth factor 23; iPTH, intact parathormone.

Table S13. Association of bone alkaline phosphatase (BAP) with different outcomes.

**BAP**		**model 1 (univariate)**	**model 2**	**model 3**
		HR [95% CI]		
**non-CV death** 387/4245	HR per SD increase	1.27 [1.16; 1.38]	1.22 [1.11; 1.33]	1.10 [0.98; 1.25]
	Q1	1 (ref.)	1 (ref.)	1 (ref.)
	Q2	1.04 [0.73; 1.47]	1.01 [0.70; 1.45]	0.90 [0.62; 1.31]
	Q3	1.25 [0.90; 1.73]	1.16 [0.82; 1.63]	1.09 [0.75; 1.59]
	Q4	1.18 [0.84; 1.65]	1.06 [0.74; 1.52]	0.97 [0.65; 1.46]
	Q5	1.78 [1.31; 2.43]	1.64 [1.18; 2.28]	1.28 [0.84; 1.95]
**CV death** 173/424	HR per SD increase	1.15 [1.00; 1.32]	1.10 [0.95; 1.28]	0.98 [0.80; 1.19]
	Q1	1 (ref.)	1 (ref.)	1 (ref.)
	Q2	1.03 [0.63; 1.68]	1.07 [0.64; 1.77]	1.02 [0.59; 1.74]
	Q3	1.12 [0.69; 1.80]	1.08 [0.66; 1.77]	1.09 [0.63; 1.88]
	Q4	1.18 [0.73; 1.90]	1.11 [0.67; 1.82]	1.05 [0.59; 1.88]
	Q5	1.27 [0.79; 2.04]	1.18 [0.71; 1.94]	0.95 [0.50; 1.79]
**MACE** 645/4244	HR per SD increase	1.13 [1.05; 1.22]	1.10 [1.01; 1.19]	1.08 [0.97; 1.20]
	Q1	1 (ref.)	1 (ref.)	1 (ref.)
	Q2	1.09 [0.85; 1.40]	1.08 [0.83; 1.40]	1.06 [0.81; 1.40]
	Q3	1.06 [0.82; 1.36]	0.95 [0.73; 1.23]	1.01 [0.76; 1.35]
	Q4	1.14 [0.89; 1.47]	1.08 [0.83; 1.40]	1.16 [0.86; 1.55]
	Q5	1.40 [1.10; 1.78]	1.27 [0.99; 1.64]	1.30 [0.94; 1.80]
**CHF** 368/4245	HR per SD increase	1.18 [1.07; 1.30]	1.08 [0.98; 1.20]	0.91 [0.79; 1.04]
	Q1	1 (ref.)	1 (ref.)	1 (ref.)
	Q2	0.94 [0.65; 1.34]	0.86 [0.59; 1.25]	0.70 [0.47; 1.04]
	Q3	1.24 [0.89; 1.74]	1.07 [0.76; 1.51]	0.94 [0.64; 1.38]
	Q4	1.45 [1.04; 2.02]	1.31 [0.93; 1.85]	1.12 [0.76; 1.67]
	Q5	1.58 [1.14; 2.19]	1.22 [0.86; 1.73]	0.91 [0.58; 1.41]

Results are presented as hazard ratios with 95%-confidence intervals given in parentheses.
Model 2: adjusted for age, sex, BMI, systolic blood pressure, LDL cholesterol, CRP, serum albumin, eGFR, UACR, diabetes mellitus, CVD, smoking, use of statins, use of RASi, use of thrombocyte aggregation inhibitors, use of betablockers, ongoing vitamin D therapy, and use of aldosterone antagonists.
Model 3: adjusted for parameters as in Model 2 plus OPG, FGF23, calcium, phosphate, P1NP, CTX1, 25-OH vitamin D, and iPTH.
Abbreviations: OPG, osteoprotegerin; HR, hazard ratio; SD, standard deviation; CV, cardiovascular; MACE, major adverse cardiac event; CHF, hospitalization due to congestive heart failure; eGFR, estimated glomerular filtration rate; UACR, urine albumin creatinine ratio; hsCRP, high-sensitivity C-reactive protein; LDL, low density lipoprotein; HDL, high density lipoprotein; BP, blood pressure; CVD, cardiovascular disease; BMI, body mass index; RASi, renin angiotensin system inhibitor; BAP, bone alkaline phosphatase; P1NP, procollagen 1 intact N-terminal propeptide; CTX1, C-telopeptide of type 1 collagen; FGF23, fibroblast growth factor 23; iPTH, intact parathormone.

Table S14. Association of C-telopeptide of type 1 collagen (CTX1) with different outcomes.

**CTX1**		**model 1 (univariate)**	**model 2**	**model 3**
		HR [95% CI]		
**non-CV death** 387/4245	HR per SD increase	1.16 [1.08; 1.25]	1.14 [1.03; 1.26]	1.01 [0.88; 1.16]
	Q1	1 (ref.)	1 (ref.)	1 (ref.)
	Q2	1.14 [0.82; 1.60]	1.07 [0.75; 1.51]	0.99 [0.69; 1.42]
	Q3	1.15 [0.82; 1.61]	1.04 [0.73; 1.47]	0.94 [0.64; 1.37]
	Q4	1.19 [0.85; 1.66]	1.00 [0.70; 1.42]	0.82 [0.55; 1.22]
	Q5	1.64 [1.20; 2.25]	1.38 [0.98; 1.97]	1.01 [0.65; 1.58]
**CV death** 173/4245	HR per SD increase	1.17 [1.04; 1.30]	1.13 [0.96; 1.32]	1.04 [0.85; 1.27]
	Q1	1 (ref.)	1 (ref.)	1 (ref.)
	Q2	0.89 [0.53; 1.49]	0.85 [0.50; 1.43]	0.86 [0.50; 1.50]
	Q3	0.98 [0.59; 1.62]	0.88 [0.52; 1.47]	0.93 [0.53; 1.65]
	Q4	1.10 [0.67; 1.78]	0.90 [0.54; 1.51]	0.97 [0.54; 1.73]
	Q5	1.70 [1.09; 2.66]	1.42 [0.86; 2.36]	1.50 [0.80; 2.83]
**MACE** 645/4244	HR per SD increase	1.09 [1.02; 1.17]	1.09 [1.00; 1.19]	1.03 [0.93; 1.15]
	Q1	1 (ref.)	1 (ref.)	1 (ref.)
	Q2	0.98 [0.76; 1.26]	0.99 [0.76; 1.29]	1.00 [0.76; 1.32]
	Q3	1.13 [0.88; 1.44]	1.13 [0.87; 1.47]	1.18 [0.89; 1.57]
	Q4	1.14 [0.89; 1.46]	1.07 [0.82; 1.40]	1.14 [0.85; 1.54]
	Q5	1.27 [1.00; 1.62]	1.26 [0.96; 1.66]	1.27 [0.90; 1.78]
**CHF** 368/4245	HR per SD increase	1.08 [0.99; 1.19]	1.13 [1.00; 1.27]	1.00 [0.86; 1.16]
	Q1	1 (ref.)	1 (ref.)	1 (ref.)
	Q2	0.87 [0.63; 1.20]	0.89 [0.64; 1.24]	0.76 [0.53; 1.08]
	Q3	0.79 [0.56; 1.10]	0.77 [0.55; 1.09]	0.65 [0.44; 0.95]
	Q4	0.96 [0.70; 1.32]	0.96 [0.69; 1.35]	0.76 [0.51; 1.12]
	Q5	1.09 [0.80; 1.48]	1.13 [0.80; 1.60]	0.78 [0.50; 1.22]

Results are presented as hazard ratios with 95%-confidence intervals given in parentheses.
Model 2: adjusted for age, sex, BMI, systolic blood pressure, LDL cholesterol, CRP, serum albumin, eGFR, UACR, diabetes mellitus, CVD, smoking, use of statins, use of RASi, use of thrombocyte aggregation inhibitors, use of betablockers, ongoing vitamin D therapy, and use of aldosterone antagonists.
Model 3: adjusted for parameters as in Model 2 plus OPG, BAP, calcium, phosphate, P1NP, FGF23, 25-OH vitamin D, and iPTH.
Abbreviations: OPG, osteoprotegerin; HR, hazard ratio; SD, standard deviation; CV, cardiovascular; MACE, major adverse cardiac event; CHF, hospitalization due to congestive heart failure; eGFR, estimated glomerular filtration rate; UACR, urine albumin creatinine ratio; hsCRP, high-sensitivity C-reactive protein; LDL, low density lipoprotein; HDL, high density lipoprotein; BP, blood pressure; CVD, cardiovascular disease; BMI, body mass index; RASi, renin angiotensin system inhibitor; BAP, bone alkaline phosphatase; P1NP, procollagen 1 Intact N-terminal propeptide; CTX1, C-telopeptide of type 1 collagen; FGF23, fibroblast growth factor 23; iPTH, intact parathormone.

Table S15. Association of serum phosphate levels with different outcomes.

Phosphate		model 1 (univariate)	model 2	model 3
		HR [95% CI]		
**non-CV death** 387/4245	HR per SD increase	0.99 [0.57; 1.73]	0.91 [0.50; 1.67]	0.93 [0.49; 1.75]
	Q1	1 (ref.)	1 (ref.)	1 (ref.)
	Q2	1.08 [0.80; 1.45]	1.10 [0.81; 1.51]	1.13 [0.82; 1.56]
	Q3	0.81 [0.59; 1.11]	0.86 [0.61; 1.20]	0.93 [0.66; 1.31]
	Q4	0.81 [0.59; 1.12]	0.91 [0.64; 1.28]	0.95 [0.66; 1.35]
	Q5	1.03 [0.76; 1.41]	1.01 [0.72; 1.42]	1.01 [0.70; 1.44]
**CV death** 173/4245	HR per SD increase	2.13 [0.95; 4.79]	1.61 [0.67; 3.89]	1.45 [0.58; 3.64]
	Q1	1 (ref.)	1 (ref.)	1 (ref.)
	Q2	0.90 [0.55; 1.46]	0.82 [0.49; 1.35]	0.88 [0.52; 1.47]
	Q3	0.96 [0.60; 1.55]	1.01 [0.62; 1.65]	1.11 [0.67; 1.84]
	Q4	0.97 [0.60; 1.57]	0.89 [0.53; 1.50]	0.89 [0.52; 1.53]
	Q5	1.32 [0.84; 2.07]	1.17 [0.71; 1.92]	1.09 [0.65; 1.83]
**MACE** 645/4244	HR per SD increase	1.75 [1.14; 2.67]	1.69 [1.05; 2.70]	1.73 [1.06; 2.81]
	Q1	1 (ref.)	1 (ref.)	1 (ref.)
	Q2	0.94 [0.73; 1.20]	0.87 [0.67; 1.12]	0.94 [0.72; 1.22]
	Q3	0.95 [0.74; 1.22]	0.94 [0.72; 1.21]	0.99 [0.76; 1.29]
	Q4	1.08 [0.84; 1.37]	1.09 [0.84; 1.41]	1.14 [0.88; 1.49]
	Q5	1.27 [1.00; 1.61]	1.22 [0.94; 1.59]	1.31 [1.00; 1.71]
**CHF** 368/4245	HR per SD increase	1.39 [0.79; 2.45]	1.59 [0.85; 2.99]	1.48 [0.77; 2.84]
	Q1	1 (ref.)	1 (ref.)	1 (ref.)
	Q2	1.11 [0.81; 1.53]	1.14 [0.81; 1.61]	1.25 [0.88; 1.77]
	Q3	0.91 [0.65; 1.27]	1.04 [0.73; 1.48]	1.19 [0.83; 1.70]
	Q4	1.14 [0.83; 1.58]	1.20 [0.84; 1.71]	1.32 [0.92; 1.89]
	Q5	1.20 [0.87; 1.66]	1.32 [0.92; 1.89]	1.31 [0.90; 1.90]

Results are presented as hazard ratios with 95%-confidence intervals given in parentheses.
Model 2: adjusted for age, sex, BMI, systolic blood pressure, LDL cholesterol, CRP, serum albumin, eGFR, UACR, diabetes mellitus, CVD, smoking, use of statins, use of RASi, use of thrombocyte aggregation inhibitors, use of betablockers, ongoing vitamin D therapy, and use of aldosterone antagonists.
Model 3: adjusted for parameters as in Model 2 plus OPG, BAP, calcium, FGF23, P1NP, CTX1, 25-OH vitamin D, and iPTH.
Abbreviations: OPG, osteoprotegerin; HR, hazard ratio; SD, standard deviation; CV, cardiovascular; MACE, major adverse cardiac event; CHF, hospitalization due to congestive heart failure; eGFR, estimated glomerular filtration rate; UACR, urine albumin creatinine ratio; hsCRP, high-sensitivity C-reactive protein; LDL, low density lipoprotein; HDL, high density lipoprotein; BP, blood pressure; CVD, cardiovascular disease; BMI, body mass index; RASi, renin angiotensin system inhibitor; BAP, bone alkaline phosphatase; P1NP, procollagen 1 intact N-terminal propeptide; CTX1, C-telopeptide of type 1 collagen; FGF23, fibroblast growth factor 23; iPTH, intact parathormone.

Table S16. Association of procollagen 1 intact N-terminal propeptide (P1NP) with different outcomes.

**P1NP**		**model 1 (univariate)**	**model 2**	**model 3**
		HR [95% CI]		
**non-CV death** 387/4245	HR per SD increase	1.1 [0.91; 1.34]	1.33 [1.08; 1.65]	1.01 [0.75; 1.35]
	Q1	1 (ref.)	1 (ref.)	1 (ref.)
	Q2	0.86 [0.62; 1.18]	0.87 [0.63; 1.22]	0.77 [0.54; 1.10]
	Q3	1.03 [0.76; 1.39]	1.14 [0.83; 1.57]	0.92 [0.64; 1.32]
	Q4	0.78 [0.56; 1.08]	0.93 [0.66; 1.31]	0.74 [0.49; 1.10]
	Q5	1.10 [0.82; 1.49]	1.36 [0.99; 1.88]	0.88 [0.57; 1.37]
**CV death** 173/4245	HR per SD increase	1.00 [0.74; 1.36]	1.37 [0.99; 1.88]	1.18 [0.76; 1.82]
	Q1	1 (ref.)	1 (ref.)	1 (ref.)
	Q2	1.02 [0.65; 1.58]	0.99 [0.63; 1.57]	0.82 [0.50; 1.35]
	Q3	0.73 [0.45; 1.19]	0.86 [0.52; 1.41]	0.70 [0.40; 1.22]
	Q4	0.78 [0.49; 1.25]	1.04 [0.64; 1.70]	0.85 [0.47; 1.52]
	Q5	0.89 [0.56; 1.40]	1.24 [0.77; 2.02]	0.90 [0.47; 1.73]
**MACE** 645/4244	HR per SD increase	0.89 [0.75; 1.04]	1.10 [0.92; 1.31]	0.86 [0.67; 1.09]
	Q1	1 (ref.)	1 (ref.)	1 (ref.)
	Q2	1.06 [0.84; 1.33]	0.96 [0.75; 1.21]	0.88 [0.68; 1.13]
	Q3	0.84 [0.66; 1.07]	0.88 [0.68; 1.13]	0.75 [0.56; 0.99]
	Q4	0.81 [0.64; 1.04]	0.92 [0.71; 1.18]	0.73 [0.54; 0.98]
	Q5	0.79 [0.62; 1.01]	1.01 [0.78; 1.31]	0.67 [0.47; 0.94]
**CHF** 368/4245	HR per SD increase	1.05 [0.86; 1.29]	1.38 [1.10; 1.73]	1.33 [0.98; 1.80]
	Q1	1 (ref.)	1 (ref.)	1 (ref.)
	Q2	1.50 [1.09; 2.06]	1.51 [1.09; 2.11]	1.49 [1.04; 2.13]
	Q3	1.06 [0.75; 1.49]	1.22 [0.85; 1.74]	1.18 [0.79; 1.78]
	Q4	1.00 [0.71; 1.42]	1.27 [0.89; 1.83]	1.24 [0.80; 1.91]
	Q5	1.30 [0.94; 1.81]	1.72 [1.21; 2.45]	1.49 [0.93; 2.38]

Results are presented as hazard ratios with 95%-confidence intervals given in parentheses.
Model 2: adjusted for age, sex, BMI, systolic blood pressure, LDL cholesterol, CRP, serum albumin, eGFR, UACR, diabetes mellitus, CVD, smoking, use of statins, use of RASi, use of thrombocyte aggregation inhibitors, use of betablockers, ongoing vitamin D therapy, and use of aldosterone antagonists.
Model 3: adjusted for parameters as in Model 2 plus OPG, BAP, calcium, phosphate, FGF23, CTX1, 25-OH vitamin D, and iPTH.
Abbreviations: OPG, osteoprotegerin; HR, hazard ratio; SD, standard deviation; CV, cardiovascular; MACE, major adverse cardiac event; CHF, hospitalization due to congestive heart failure; eGFR, estimated glomerular filtration rate; UACR, urine albumin creatinine ratio; hsCRP, high-sensitivity C-reactive protein; LDL, low density lipoprotein; HDL, high density lipoprotein; BP, blood pressure; CVD, cardiovascular disease; BMI, body mass index; RASi, renin angiotensin system inhibitor; BAP, bone alkaline phosphatase; P1NP, procollagen 1 intact N-terminal propeptidel; CTX1, C-telopeptide of type 1 collagen; FGF23, fibroblast growth factor 23; iPTH, intact parathormone.

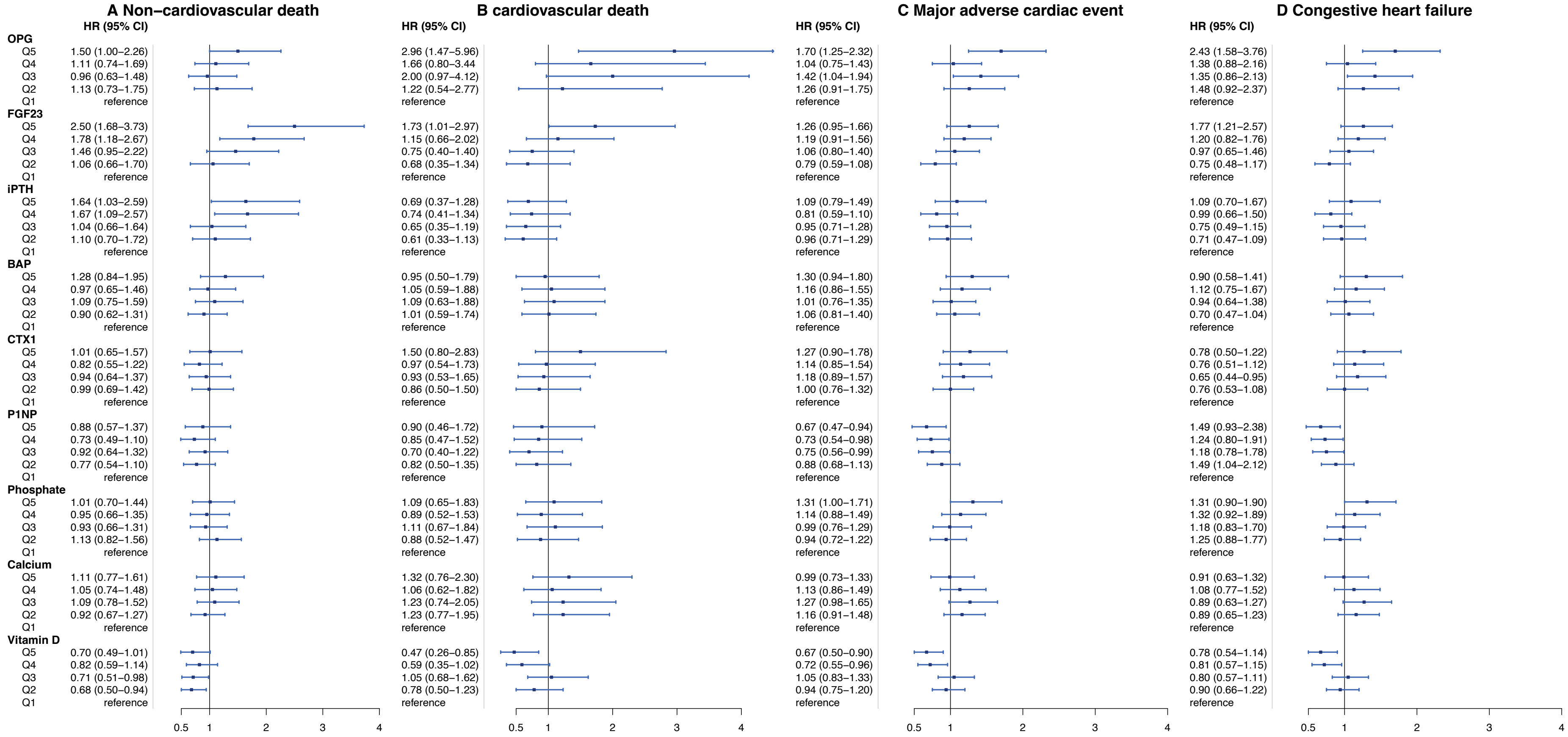

Supplementary Figure S1. Risk of outcomes for each biomarker quintile in model 3.

Here presented data is the output of model 3 which adjusts for demographic characteristics and the other bone biomarkers. CV, cardiovascular; HR, hazard ratio; CI, confidence interval; MACE, major adverse cardiac event; CHF, hospitalization due to congestive heart failure; OPG, osteoprotegerin; iFGF23, intact fibroblast growth factor 23; iPTH, intact parathyroid hormone; BAP, bone alkaline phosphatase; CTX1, cross-linked C-telopeptide of type I collagen; P1NP, procollagen I intact N-terminal propetide.

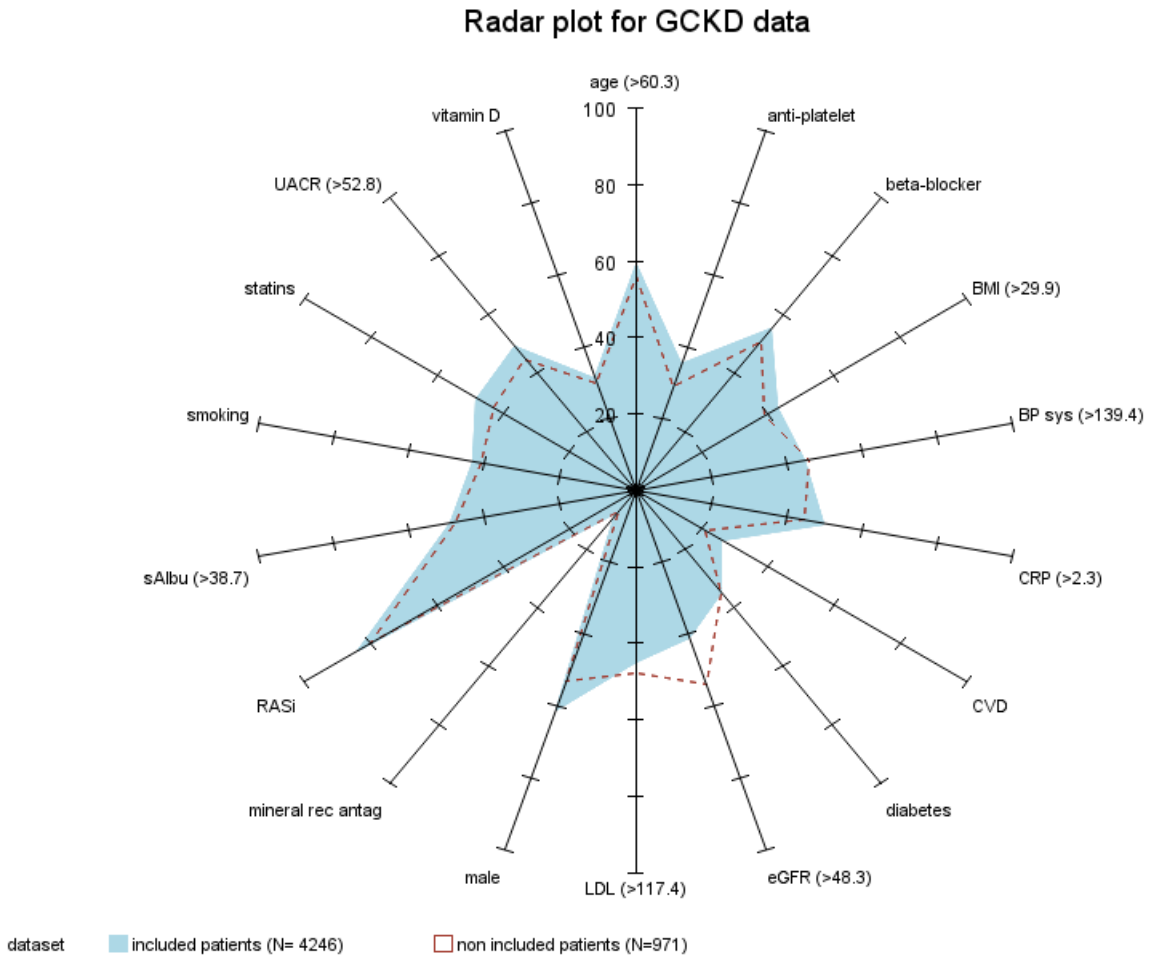

Supplementary Figure 2. Radar plot for GCKD study data. The participants included in the analysis (highlighted in blue) had comparable characteristics to those which were not included into the analysis.

Abbreviations: BP sys, systolic blood pressure; CRP, high-sensitivity C-reactive protein; CVD, cardiovascular disease; eGFR, estimated glomerular filtration rate; LDL, low density lipoprotein cholesterol; mineral rec antag, mineral corticoid receptor antagonist; RASi, renin-angiotensin-system inhibitors; sAlbu, serum albumin; UACR, urine albumin creatinine ratio.
